# PacBio and Illumina RNA Sequencing Identify Alternative Splicing Events in Response to Cold Stress in Two Poplar Species

**DOI:** 10.3389/fpls.2021.737004

**Published:** 2021-10-07

**Authors:** Jingli Yang, Wanqiu Lv, Liying Shao, Yanrui Fu, Haimei Liu, Chengjun Yang, Aihua Chen, Xieyu Xie, Zhiwei Wang, Chenghao Li

**Affiliations:** ^1^State Key Laboratory of Forest Genetics and Tree Breeding, Northeast Forestry University, Harbin, China; ^2^Biology Group, Jiamusi No.1 High School, Jiamusi, China; ^3^Economic Forest Laboratory, Mudanjiang Branch of Heilongjiang Academy of Forestry, Mudanjiang, China

**Keywords:** *Populus trichocarpa*, *Populus ussuriensis*, alternative splicing, cold stress, PacBio, RNA-Seq

## Abstract

In eukaryotes, alternative splicing (AS) is a crucial regulatory mechanism that modulates mRNA diversity and stability. The contribution of AS to stress is known in many species related to stress, but the posttranscriptional mechanism in poplar under cold stress is still unclear. Recent studies have utilized the advantages of single molecular real-time (SMRT) sequencing technology from Pacific Bioscience (PacBio) to identify full-length transcripts. We, therefore, used a combination of single-molecule long-read sequencing and Illumina RNA sequencing (RNA-Seq) for a global analysis of AS in two poplar species (*Populus trichocarpa* and *P. ussuriensis*) under cold stress. We further identified 1,261 AS events in *P. trichocarpa* and 2,101 in *P. ussuriensis* among which intron retention, with a frequency of more than 30%, was the most prominent type under cold stress. RNA-Seq data analysis and annotation revealed the importance of calcium, abscisic acid, and reactive oxygen species signaling in cold stress response. Besides, the low temperature rapidly induced multiple splicing factors, transcription factors, and differentially expressed genes through AS. In *P. ussuriensis*, there was a rapid occurrence of AS events, which provided a new insight into the complexity and regulation of AS during cold stress response in different poplar species for the first time.

## Introduction

In eukaryotes, precursor messenger RNAs (pre-mRNAs) with multiple introns undergo alternative splicing (AS) to generate two or more mature mRNA isoforms, encoding structurally and functionally different proteins (Palusa et al., 2007). AS is a crucial regulatory mechanism that contributes to cellular and functional complexity and has been extensively studied in animals and plants (Wang et al., 2018; Li et al., 2019). Genome-wide investigation of AS has been performed on development or in response to stresses in multiple plants. Studies have revealed that an estimated 60% of Arabidopsis, 50% of soybean, 40% of cotton, and 40% of maize intron-containing genes undergo AS (Marquez et al., 2012; Syed et al., 2012; Li et al., 2019). Five major types of AS events are recognized, namely exon skipping (ES), intron retention (IR), mutually exclusive exon (MXE), alternative 5′ splice site (A5SS), and alternative 3′ splice site (A3SS), of which IR is the most common event in plants (Reddy et al., 2013). Evidence suggests that plants employ AS to achieve phenotypic plasticity in response to different abiotic stresses, such as heat, drought, salinity, and cold (Zhao et al., 2014; Liu et al., 2018; Zhu et al., 2018).

Several studies have identified and reported the complexity of AS in plant species, such as cotton (Wang et al., 2018), cassava (Li et al., 2019), *Phyllostachys edulis* (Wang et al., 2017), *Populus trichocarpa* (Bao et al., 2013), Arabidopsis (Marquez et al., 2012), and rice (Zhang et al., 2019). Different from the constitutively spliced isoforms, alternatively spliced ones always show cell-, tissue-, or condition-specific expression patterns, and the extent of AS in plants depends on the complexity of tissues (Kalsotra and Cooper, 2011). Gan et al. (2011) identified multiple varied AS events among the different genotypes of *Arabidopsis thaliana*. Zhou et al. (2011) discovered a total of 45 AS events in *Brassica oleracea* in two organs and under two environmental conditions (heat and cold), while the splice sites in the pre-mRNA are recognized by splicing factors (SFs), which recruit the splice osome for intron removal, to generate different alternatively spliced isoforms (Fu and Ares, 2014; Lee and Rio, 2015). Besides, SFs are essential for plant growth and development processes, including control of flowering time, regulation of the circadian rhythms, and response to abiotic stresses (Staiger and Brown, 2013; Schlaen et al., 2015). These studies indicate that the regulated AS of downstream targets is essential for plants. A recent survey by Calixto et al. (2018) has reported a massive and rapid AS response under cold stress that involved changes in hundreds of cold-responsive transcription factors (TFs) and SFRNA-binding proteins in *Arabidopsis*. Moreover, misexpression of SFs altered cold sensitivity or tolerance in plants (Reddy et al., 2013; Staiger and Brown, 2013). In Arabidopsis, low temperature regulated the expression and splicing patterns of the serine/arginine-rich SFs (Palusa et al., 2007). These studies strongly suggest the central role of SFs and the importance of AS in plant response to cold stress.

Studies have widely used short-read RNA sequencing (RNA-Seq) technology to detect AS events; however, it is challenging to identify full-length splicing isoforms accurately by this method. By contrast, the SMRT developed by Pacific Biosciences (PacBio, CA, USA) offers an improvement in read length over the previous technologies. PacBio technology is valuable for the annotation of newly sequenced genes and the analysis of AS (Eid et al., 2009; Sharon et al., 2013). Researchers have started using PacBio sequencing technology to characterize the complexity of transcriptome in various plant species.

Low temperature is one of the most common stresses that negatively affect plant growth and crop production (Calixto et al., 2018). Numerous studies have demonstrated changes in AS events in response to external cold/chilling stimuli; however, the regulation of AS in poplar species under cold stress is not clear at the whole transcriptomic level. In this study, it was better cold tolerance in *P. ussuriensis* than in *P. trichocarpa*. We aim to investigate the reason for this. Our data reveal that cold intensely affects gene expression *via* alternative splicing regulated by SFs in *P. ussuriensis* to resistant cold stress and advances our understanding of the high complexity and specificity of species-specific AS regulation in response to cold.

## Materials and Methods

### Plant Materials and Growth Conditions

**Figure 1:**
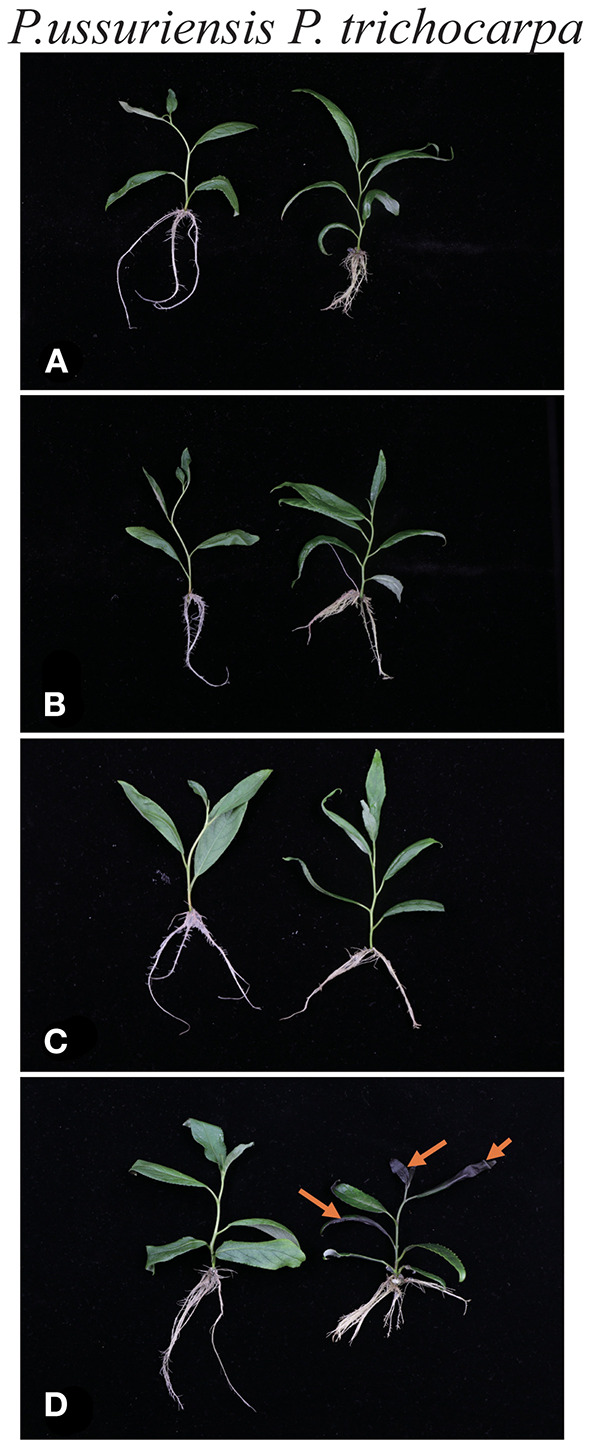
Phenotypic changes in *P. trichocarpa* and *P. ussuriensis* in response to cold stress. *P. trichocarpa* and *P. ussuriensis* grown at 25°C for 3 h **(A)**; 3°C for 3h **(B)**; −3°C for 3 h **(C)**; and −3°C for 4 h **(D)**.

*Populus trichocarpa* “Nisqually-1” and *P. ussuriensis* Kom. were used in this study. Stem segments (3 cm long) of these poplar species were cultured in ½ MS medium for 1 month. These clonally cultured seedlings were placed at 25 ± 2°C under 16-h/8-h (light/dark) photoperiod. Both 2-week-old plants were transferred to a chamber for cold treatment at 3 and −3°C for 3 h, respectively. The young leaves and shoot apices of the control sample (25°C), 3°C, and −3°C treated samples were collected at the same time and immediately frozen in liquid nitrogen and stored at −80°C for RNA extraction. Each time point was repeated with three biological replicates. The nine RNAs samples (25, 3, and −3°C with three replicates) from each poplar species were subjected to 150 bp paired-end sequencing using the Illumina HiSeq platform, respectively. The nine RNAs samples from each poplar species were mixed in equal volume and sequenced on the PacBio RS II platform, respectively (Figure 1A).

### RNA Extraction and the SMRT Sequencing Library Construction

Total RNA was extracted from the leaves and shoot apices of *P. trichocarpa* and *P. ussuriensis* maintained under different temperatures (25, 3, and −3°C) using the CTAB method (Jaakola et al., 2001). The total RNA was assessed using Agilent Bioanalyzer 2100 (Agilent, https://www.agilent.com). RNA purity was checked using the kaiao K5500® Spectrophotometer (Kaiao, Beijing, China). RNA integrity and concentration were assessed using the Bioanalyzer 2100 RNA Nano 6000 Assay Kit (Agilent Technologies, CA, USA). Total RNA of the nine samples from each species was pooled in equal amounts, and 1 μg of the pooled RNA was used for cDNA synthesis and SMRTbell library construction. The purified RNA was reversely transcribed into cDNA using the SMARTer PCR cDNA Synthesis Kit (Clontech Laboratories, Inc. Mountain View, CA, USA). The cDNA was amplified using the Kapa HiFi PCR Kit (Kapa Biosystems, Wilmington, MA, USA). Size selection was carried out on a BluePippin (Sage Science, Beverly, MA, USA), and 1–2, 2–3, 3–6, and 5–10 kb fractions were collected. These collected cDNA fractions were treated with a DNA damage repair mix, followed by end repair and ligation of SMRT adapters using the PacBio SMRTbell Template Prep Kit (Pacific Biosciences, Menlo Park, CA, USA) to create SMRT sequencing libraries.

### PacBio Data Analysis

Raw data generated by the PacBio RSII platform were processed following the Iso-seq method. The PacBio raw reads were processed into error-corrected “reads of inserts” (ROIs) using ToFu (version 2.3.0) (Gordon et al., 2015) with the following parameters: minimum full pass, >1; minimum ROI length, >200 nucleotides; and prediction accuracy, >0.8. The ROIs were classified into circular consensus sequences (CCS) and non-CCS subreads based on the presence or absence of sequencing adapters. CCS subreads were classified into full-length non-chimeric reads (FLNC) or non-FLNC based on the presence of both the primer sequences (the 5′ and 3′ sequences) and the polyA tail signal.

Next, isoform-level clustering algorithm ICE (Iterative Clustering for Error Correction) (Gordon et al., 2015) was applied to all the full-length (FL) transcripts to obtain the consensus transcripts based on the sequence similarity and generate a consensus sequence for each cluster. Quiver was used for error correction to obtain high-quality (HQ) and low-quality (LQ) isoforms (accuracy, ≥99%). Finally, high-quality FL transcripts were combined to obtain all high-quality FL transcripts of each sample.

### Library Preparation and Illumina Sequencing

Illumina sequencing was performed to generate data that can be used to validate and quantify the PacBio transcripts. Approximately, 2 μg of RNA per sample was used as input material for the Illumina sequencing. The clustering of the index-coded samples was performed on a cBot cluster generation system (IlluminaHiSeq PE Cluster Kit v4-cBot-HS), following the instructions of the manufacturer. After cluster generation, the libraries were sequenced on an Illumina platform, and 150 bp paired-end reads were generated. In order to guarantee the data quality, the original data (raw data) were filtered. The read counts for each gene in each sample were generated using HTSeq v0.6.0, and FPKM (fragments per kilobase million mapped reads) values were then calculated to estimate the expression level of the genes in each sample. Gene annotation was based on the following databases: Nr (NCBI nonredundant protein sequences), Nt (NCBI nonredundant nucleotide sequences), Swiss-Prot (a manually annotated and reviewed protein sequence database), Pfam (protein family), COG (clusters of orthologous groups of proteins), GO (gene ontology), KEGG (Kyoto Encyclopedia of Genes and Genomes) orthology database (Kanehisa et al., 2008), and GO enrichment analysis of the differentially expressed genes (DEGs) were implemented by the GOseq (Young et al., 2010). Genes with *q* ≤ 0.05 and |log2_ratio| ≥ 1 are identified as DEGs.

### Identification of Differentially Spliced Events

The tool ASprofile (version b-1.0.4) was employed to classify the AS events using the raw.gtffiles assembled from the Illumina RNA-Seq and SMRT-Seq data of *P. ussuriensis* compared with *P. trichocarpa*. The AS events, including IR, ES, MXE, A5SS, and A3SS, were extracted and counted. The differential alternative splicing (DAS) events from two poplar comparisons at different temperatures were identified by rMATS.3.2.2 (Shen et al., 2014). The AS events with a false discovery rate (FDR) <0.05 were defined as DAS events. GO enrichment analyses with DAS were conducted using AgriGO (Tian et al., 2017).

### Calcium and Physiological Indicators Determination

After cold stress, the leaves of 25, 3, −3°C for *P. trichocarpa* and *P. ussuriensis* were collected and dried at 70°C in an oven. Approximately 0.5 mg (dry weight) of samples were weighted and analyzed using an inductively coupled plasma (ICP) emission spectrometer (ICP-OES 5110 VDV, Agilent, USA). These data were averaged from three replicates (*n* = 3).

For MDA content measurement, about 50 mg of leaves were ground and homogenized in 1 ml of 0.1% (w/v) TCA for 10 min and centrifuged at 10,000 *g* for 15 min at 4°C. The supernatant was reacted with 20% (w/v) TCA, containing 0.5% (w/v) thiobarbituric acid. After being boiled and cooled, it was centrifuged at 10,000 *g* for 5 min at 4°C. The detailed method was performed as described by Metwally et al. (2003). These data were averaged from three replicates (*n* = 3).

The H_2_O_2_ was measured using an Amplex Red Hydrogen Peroxide/Peroxidase Assay Kit (Invitrogen, Carlsbad, CA, USA) as described in Xing et al. (2008). Leaves of poplars were frozen in N_2_ and ground. The phosphate buffer (20 mM K_2_HPO_4_, pH6.5) was added to 50 mg of ground frozen tissue. After centrifugation, the supernatant was incubated with 0.2-U ml^−1^ horseradish peroxidase and 100-μM Amplex Red reagent (10-acetyl-3,7-dihydrophenoxazine) at room temperature for 30 min in darkness. The fluorescence was quantified using FLUOStarOptima (excitation at 560 nm and emission at 590 nm). These data were averaged from three replicates (*n* = 3).

### Validation of Alternative Splicing Transcripts

Total RNA was extracted from the leaves and shoot apices of *P. ussuriensis* as described above, which was consistent with transcriptome sequencing. The TransScript One-Step gDNA Removal and cDNA Synthesis SuperMix Kit (TransGen Biotech, China) was used for simultaneous genomic DNA removal and cDNA synthesis (20 μl) following the instructions of the manufacturer. The AS transcripts were validated by reverse transcription-PCR (RT-PCR) using specific primers listed in Supplementary Table S1.

### Quantitative Real-Time PCR (qRT-PCR) Analysis

Total RNA extraction and cDNA synthesis were performed as described above. QRT–PCR was performed to validate the expression level of DEGs, DAS isoforms that respond to cold stress, DAS of splicing-associated factors, and TFs. The *PuActin* gene was used as the internal control. The relative gene expression values in *P. ussuriensis* were analyzed using the 2^−ΔΔCt^ method compared with *P. trichocarpa*. All reactions were performed using three biological replicates for each sample. The primers are listed in Supplementary Tables S2–S5.

### Statistical Analysis

All statistical analyses were performed using SPSS software version 20 (IBM, Chicago, USA), and significant differences were evaluated using analysis of variance (ANOVA). Statistically significant differences were indicated by ^*^p < 0.05 and ^**^p < 0.01.

## Results

### Phenotypic Changes in Two Poplar Species in Response to Cold Stress

Low temperature is one of the key environmental stresses, which negatively impairs plant growth and development (Li et al., 2017). To evaluate the tolerance of cold, we compared the phenotypic changes of two poplar species *P. trichocarpa* and *P. ussuriensis*, which are tolerant and sensitive to cold stress, respectively. About 5 cm height of seedlings was used for physiological and transcriptome analyses at 3°C for 3 h and −3°C for 3 h. The seedlings cultured at room temperature (Figure 1A) were used as control. Treatment-related changes in the phenotype of *P. trichocarpa* were observed after 4 h at −3°C (Figure 1D); the leaves were damaged and wilted, while the *P. ussuriensis* plants were not damaged (Figure 1D). In comparison, no difference was observed after 3 h at 3°C (Figure 1B) and −3°C (Figure 1C) between the two species.

### Calcium, MDA, POD, and H_2_O_2_ Determination

**Figure 2:**
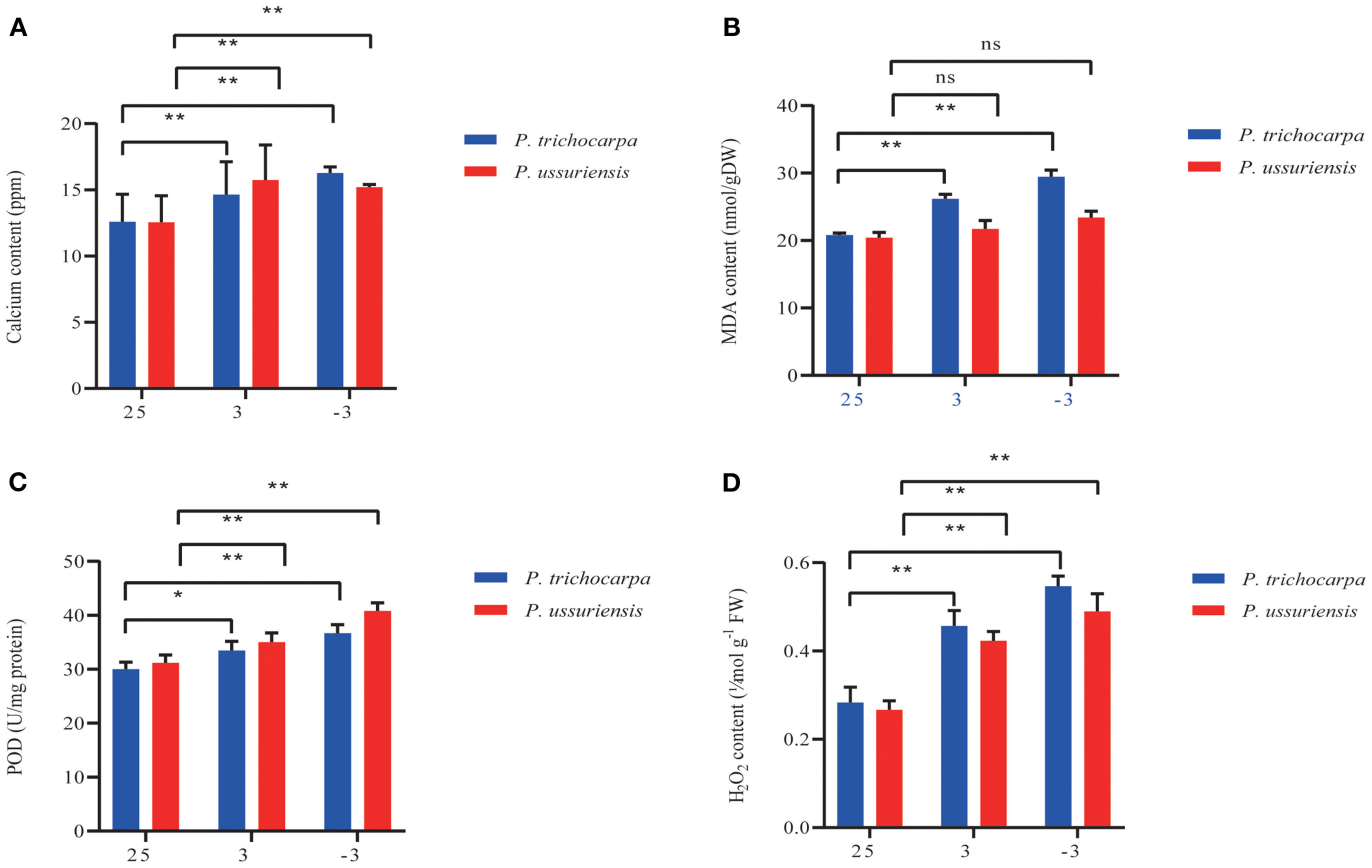
Calcium and physiological indicators measurement in *P. trichocarpa* and *P. ussuriensis*. **(A)** Calcium content measurement using inductively coupled plasma (ICP) emission spectrometer (ICP-OES 5110 VDV, Agilent, USA). **(B)** MDA, **(C)** POD, and **(D)** H_2_O_2_ content measurement. *P < 0.05; ^**^P < 0.01. ns denotes no significance.

At room temperature, the content of calcium, MDA, POD, and H_2_O_2_ in *P. ussuriensis* was not different from them in *P. trichocarpa* (Figure 2). As the temperature fell, the calcium (Figure 2A), MDA (Figure 2B), POD (Figure 2C), and H_2_O_2_ (Figure 2D) content in both poplars was increased. But the calcium (Figure 2A), and POD (Figure 2C) content in *P. ussuriensis* increased more than that in *P. trichocarpa*, while the MDA (Figure 2B) and H_2_O_2_ (Figure 2D) content in *P. ussuriensis* decreased more than that in *P. trichocarpa* at 3 and −3°C.

### Overview of PacBio Iso-Seq Data of Two Poplar Species

**Figure 3:**
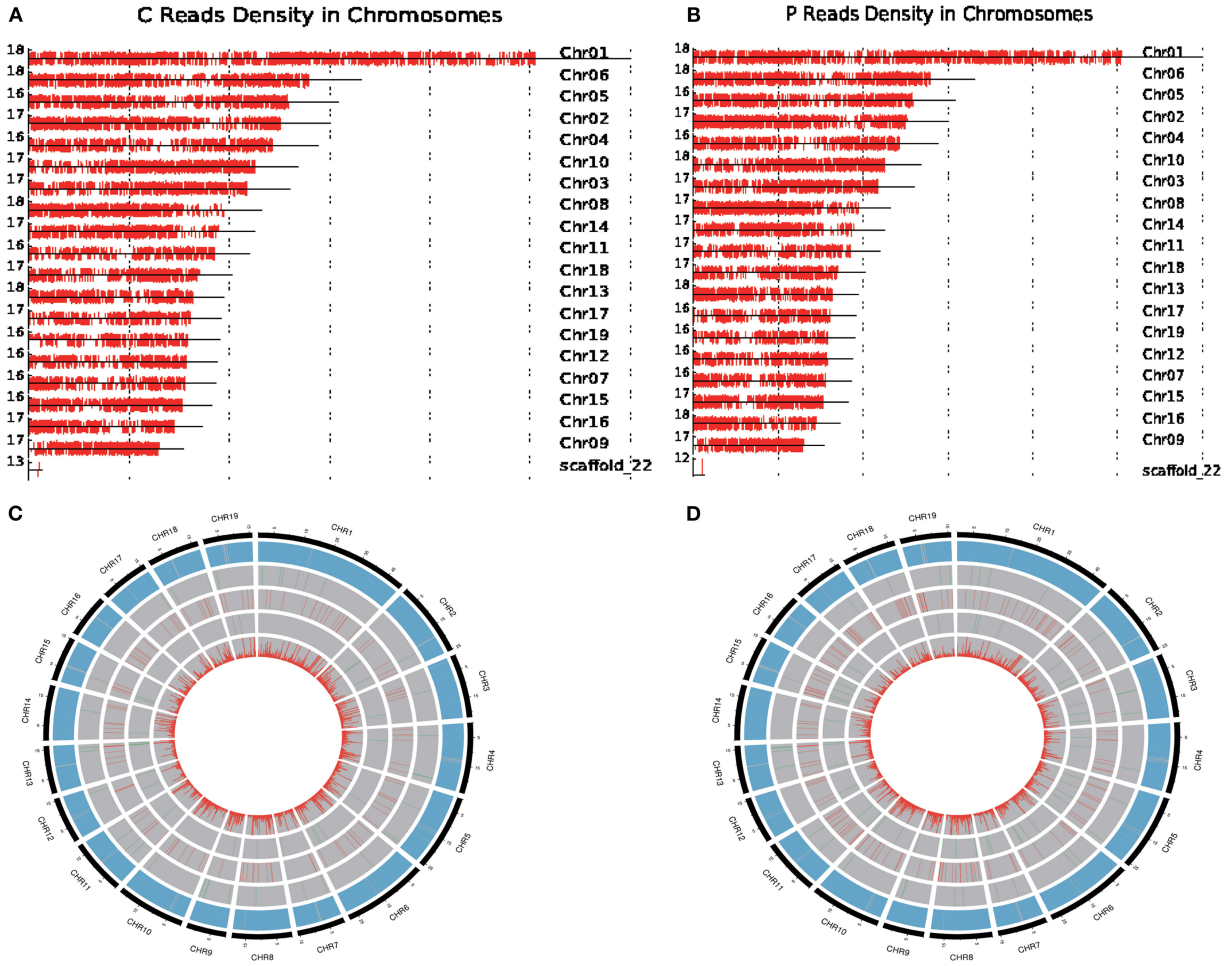
Circos visualization of PacBio Isoseq data in *P. trichocarpa* and *P. ussuriensis* compared using SMRT sequencing. **(A,B)** Distribution of full-length reads density in *P. trichocarpa*. and *P*. ussuriensis, respectively, on reference chromosome, respectively. **(C,D)** The distribution of genetic variants in *P. trichocarpa*. and *P. ussuriensis*, respectively.

We used *P. ussuriensis*, the widely cultivated species in northeast China, to compare with *P. trichocarpa* for this study. We performed PacBio sequencing and conduce analysis based on *P. trichocarpa* genome. From the phenotypic point of view, *P. ussuriensis* was more tolerant to lower temperatures than *P. trichocarpa*. To better understand the full-length splice variants, novel genes, and alternative polyadenylation (APA) sites of the two species in response to cold stress, the PacBio Iso-Seq method was used to sequence the transcriptomes. A total of two SMRT cells (r64053_20191003_023517_4_H01 for *P. trichocarpa*; r64053_20191003_023517_4_H01 for *P. ussuriensis*) mixed with nine samples were constructed to eliminate instrumental bias toward short fragments, respectively. A dataset with more than 20 Gb of clean reads was obtained after filtering using SMRTLink (6.0). Referring to *P. trichocarpa* genome, a total of 51,153 and 58,082 consensus isoforms were obtained from the two libraries (*P. trichocarpa* and *P. ussuriensis*), respectively (Supplementary Table). CCS reads were self-corrected to obtain high-quality (HQ) reads of insert (ROI) when minimum full pass, >1; minimum ROI length, >200 nt; and prediction accuracy, >0.8 for adaptors. Then, a clustering algorithm, isoform-level clustering algorithm (ICE), was applied to all full-length transcripts to generate the consensus transcripts. It grouped them into clusters based on the sequence similarity and generated a consensus isoform for each cluster. Subsequently, the untested complete insert sequence was aligned back to the consensus isoform, and then corrected to obtain HQ isoforms and low-quality (LQ) isoforms. Due to the removal of a lot of redundancy, HQ isoforms were significantly reduced compared to CCS reads. The mean length of consensus isoforms in the two libraries was between 1,683 and 1,910 bp, respectively (Table 1). A total of 50,910 reads of HQ isoforms with a mean length of 1,678 bp were identified from the library of *P. trichocarpa* (Table 1). A total of 57,872 reads of HQ isoforms with a mean length of 1,905 bp were identified from the library of *P. ussuriensis* (Table 1). Among which, a total of 228 and 188 low-quality LQ isoforms with a mean length of 1,157 and 1,344 bp were obtained, respectively (Table 1). For SMRT-seq analysis, with a total of 26,153,342 subreads, 330,371 CCS reads, and 35,414 consensus reads appeared after the modification of a site mismatch (Table 2). Mapping to the reference genome of *P. trichocarpa* RSEM (1.2.31) software (Li and Dewey, 2011), a total of 97.66 and 97.51% of the isoforms detected by PacBio SMRT-Seq were mapped reads in *P. trichocarpa* (Figure 3A) and *P. ussuriensis* (Figure 3B), respectively; 1.8 and 1.02% were multi-mapped to the genome in *P. trichocarpa* (Figure 3A) and *P. ussuriensis* (Figure 3B), respectively. The mapped isoforms (density) were spread across different chromosomes.

**Table 1:** Difference in genes as annotated in two poplar species compared with *P. trichocarpa* genome by the PacBio sequencing data, respectively.

	***P. trichocarpa***	***P. ussuriensis***
Cell_ID	r64053_20191003_023517_4_H01	r64053_20191003_023517_4_H01
Number of consensus isoforms	51,153	58,082
Mean length of consensus isoforms	1,683.17	1,910.19
Number of high-quality isoforms	50,910	57,872
Mean length of high-quality isoforms	1,678.8	1,905.13
Number of low-quality isoforms	228	188
Mean length of low-quality isoforms	1,157.82	1,344.64

**Table 2:** Statistical analysis of circular consensus sequences (CCS).

**Sample**	**Cell_ID**	**ReadsNum**	**BasesNum**	**MeanLen**	**MaxLen**	**GC (%)**	**N50**	**MeanPass**
*P. trichocarpa*	r64053_20191003_023517_4_H01	575481	999615809	1,737.01	18,761	43.4	2,387	48.37
*P. ussuriensis*	r64053_20191003_023517_4_H01	619386	1175864377	1,898.44	20,797	42.09	2,439	43.78

Furthermore, we compared the genome-wide genetic variants, including chromosome distribution, gene density, novel gene, novel long noncoding RNA (lncRNA), APA site, and single-nucleotide polymorphism (SNP), between *P. trichocarpa* and *P. ussuriensis* (Figure 3) using the Circos program. A total of 852 (Figure 3C) and 1,093 (Figure 3D) novel genes and 25 (Figure 3C) and 42 (Figure 3D) APA sites were detected in *P. trichocarpa* and *P. ussuriensis*, respectively. A total of 235,900 (Figure 3C) and 49,781 (Figure 3D) SNPs were detected in *P. trichocarpa* and *P. ussuriensis*, respectively.

### Identification of Known and Novel Alternative Splicing (AS) Events by SMRT-Seq

**Figure 4:**
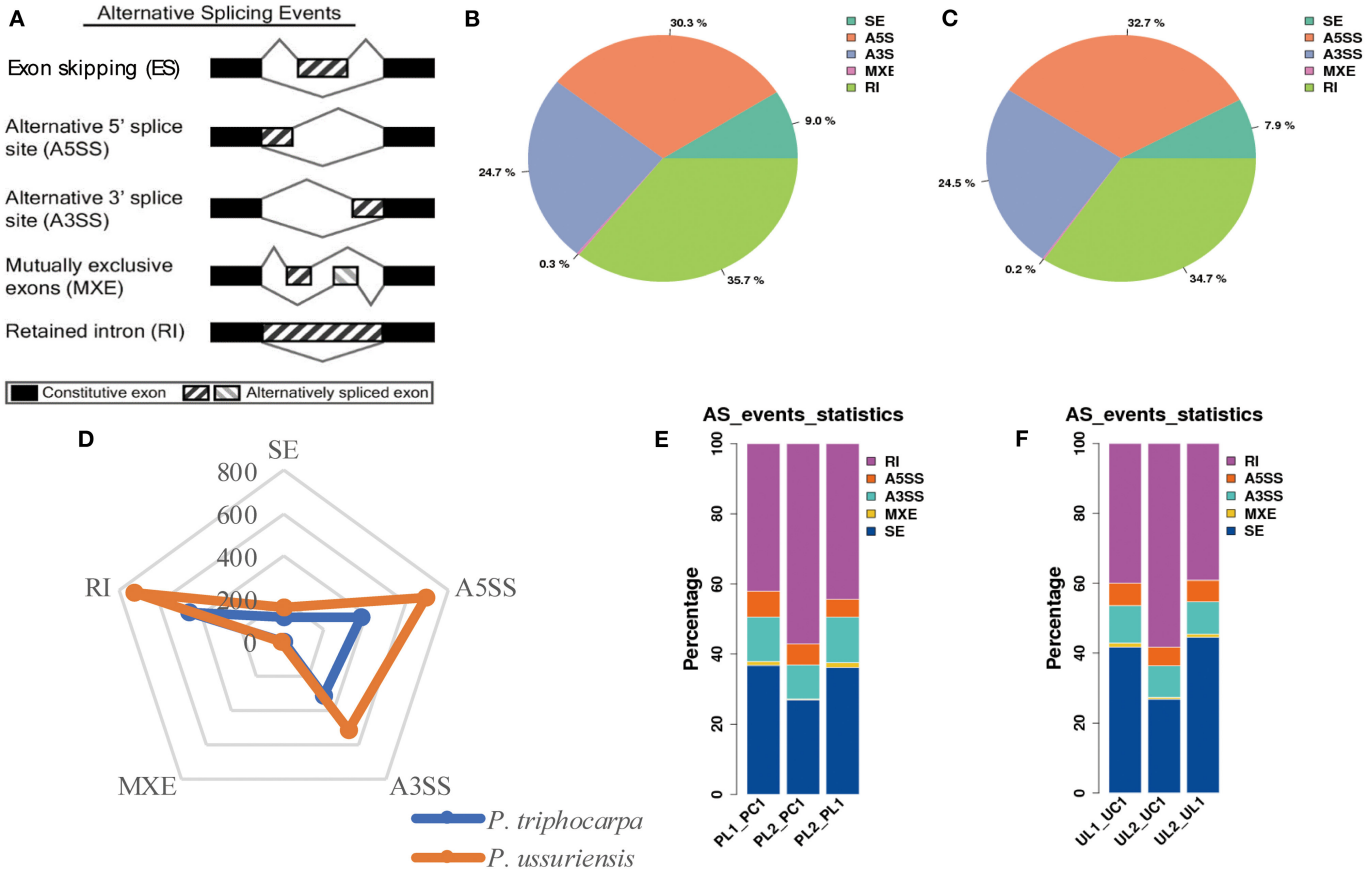
Comparison of alternative splicing (AS) events in response to cold stress in *P. trichocarpa* and *P. ussuriensis* identified by PacBio sequencing. **(A)** AS types. **(B)** The proportion of different AS types in response to cold stress in *P. trichocarpa*. **(C)** The proportion of different AS types in response to cold stress in *P. ussuriensis*. **(D)** A radar plot showing the percentage of AS types in response to cold stress in *P. trichocarpa* and *P. ussuriensis*. **(E)** Comparison of the AS types at different cold temperatures in *P. trichocarpa*. PL1_PC1: AS percentage statistical analysis at 3°C compared with 25°C in *P. trichocarpa*; PL2_PC1: AS percentage statistical analysis at −3°C compared with 25°C in *P. trichocarpa*; PL2_PL1: AS percentage statistical analysis at −3°C compared with 3°C in *P. trichocarpa*. **(F)** Comparison of the AS types at different cold temperatures in *P. ussuriensis*. UL1_UC1: AS percentage statistical analysis at 3°C compared with 25°C in *P. ussuriensis*; UL2_UC1: AS percentage statistical analysis at −3°C compared with 25°C in *P. ussuriensis*; UL2_UL1: AS percentage statistical analysis at −3°C compared with 3°C in *P. ussuriensis*.

To investigate the role of AS in response to cold stress, we surveyed the transcript isoforms in the two poplar species. We examined the five major types of AS events (IR, ES, MXE, A5SS, and A3SS) in the isoforms using SMRT-Seq (Figure 4A). We compared the frequency of AS types from the two poplar species at 25, −3, and 3°C, respectively. Based on the reference genome (*P. trichocarpa*), 35.7% of the AS events were IR, 30.3% were A5SS, 24.7% were A3SS, 9.% were ES, and 0.3% were MXE in *P. trichocarpa* (Figure 4B) while 34.7% of the AS events were IR, 32.7% were A5SS, 24.5% were A3SS, 7.9% were ES, and 0.2% were MXE in *P. ussuriensis* (Figure 4C). The number of AS events in *P. ussuriensis* under cold stress was more than that in *P. trichocarpa* (Figure 4D). Among the AS types, IR, with a frequency of more than 30%, was the most abundant type under cold stress (Figure 4E). Interestingly, the percentage of different AS types changed with a decrease in temperature in both poplar species, especially IR dramatically increased and ES decreased at −3°C compared with that at 3°C both in *P. trichocarpa* and *P. ussuriensis* (Figures 4E,F).

### Analysis of DEGs, Differential Alternative Splicing (DAS) Events, and Corresponding Cold-Responsive Genes by RNA-Seq

**Figure 5:**
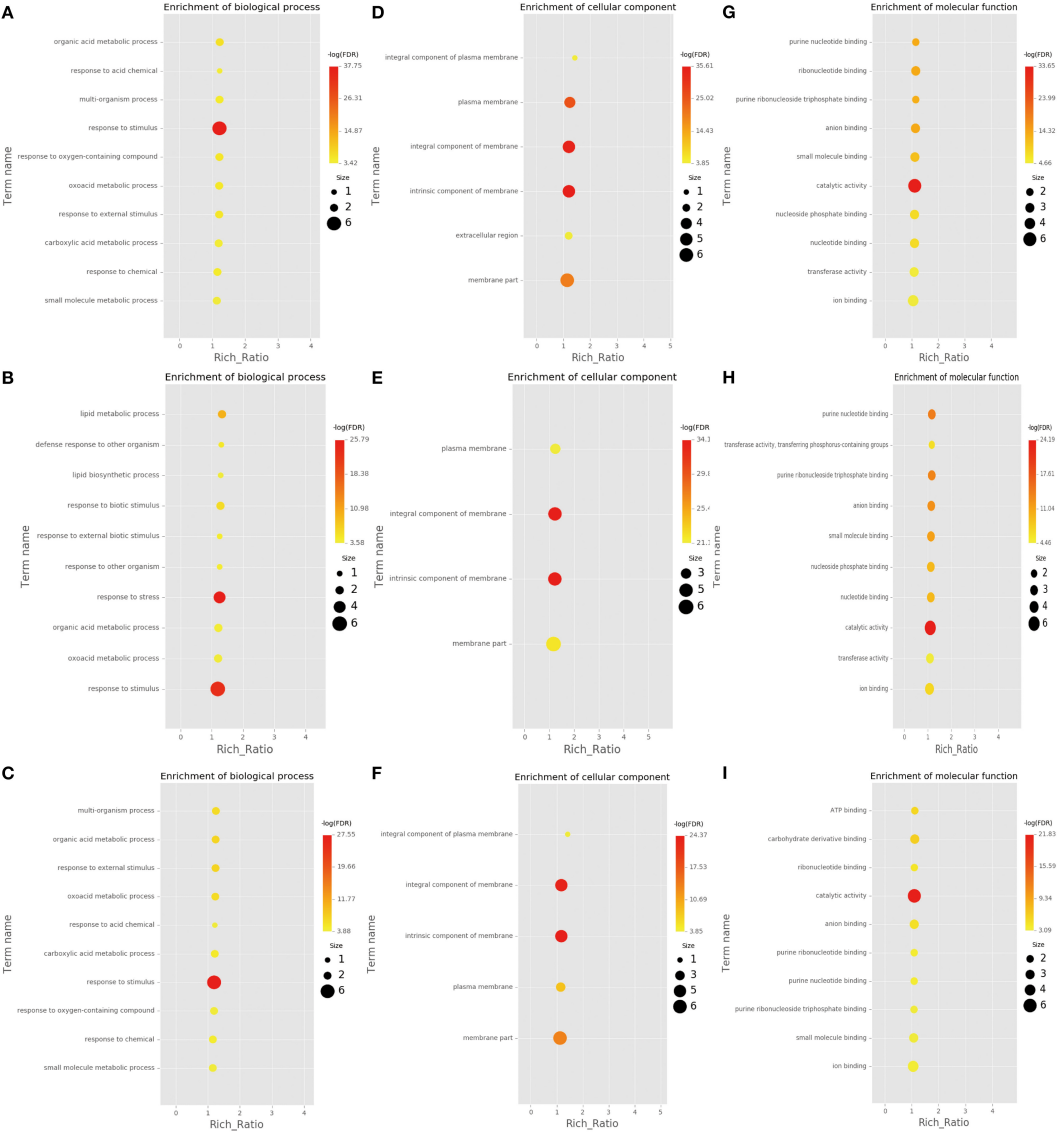
Most significantly enriched gene ontology (GO) terms for differentially expressed genes (DEGs) identified by RNA sequencing analysis of two poplar species under cold conditions. **(A–C)** GO terms for DEGs between *P. ussuriensis* and *P. trichocarpa* at 25°C. **(D–F)** GO terms for DEGs between *P. ussuriensis* and *P. trichocarpa* at 3°C. **(G–I)** GO terms for DEGs between *P. ussuriensis* and *P. trichocarpa* at −3°C.

**Figure 6:**
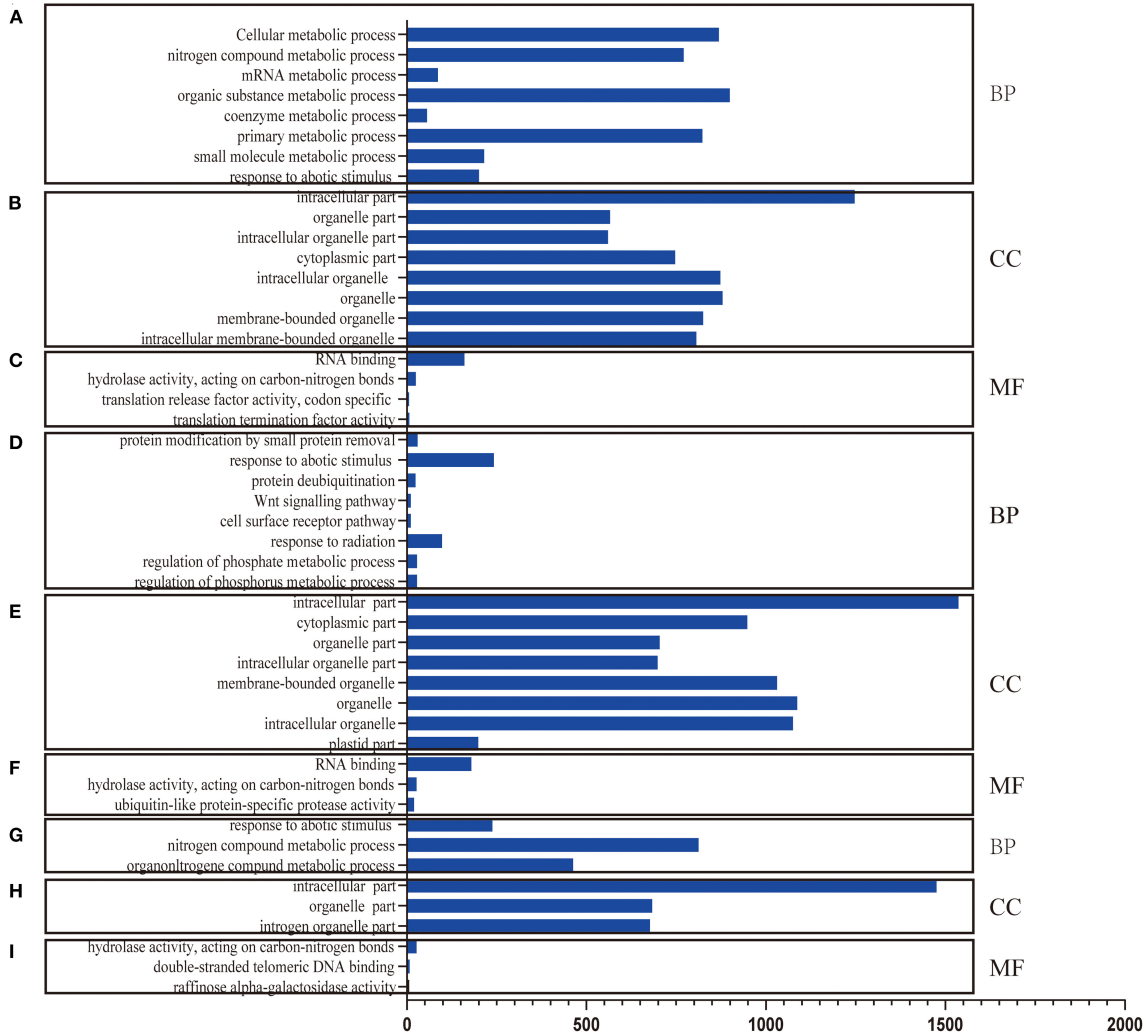
Differential alternative splicing (DAS) genes identified by RNA sequencing analysis of two poplar species under cold conditions. **(A–C)** GO terms for DAS genes between *P. ussuriensis* and *P. trichocarpa* at 25°C. **(D–F)** GO terms for DAS genes between *P. ussuriensis* and *P. trichocarpa* at 3°C. **(G–I)** GO terms for DAS genes between *P. ussuriensis* and *P. trichocarpa* at −3°C.

RNA-Seq helps understand the network *via* AS influence gene expression and transcriptome reprogramming. To improve the accuracy of AS analysis, we investigated the transcriptional changes and compared the two poplar species in response to cold stress by RNA-seq. Compared to *P. trichocarpa*, 6,060 (Supplementary Data S1), 5,208 (Supplementary Data S2), and 5,867 (Supplementary Data S3) DEGs (*p* < 0.05;|log2 (FC)|>1) were identified at 25, 3, and −3°C, respectively, in *P. ussuriensis*. We further analyzed the most significantly enriched GO terms for DEGs (Figures 5A–I) and DAS (Figures 6A–I) genes. The most significantly enriched GO terms for DEGs were “response to stimulus and stress” in biological process (BP, Figures 5A–C), an “intrinsic component of membrane” in a cellular component (CC, Figures 5D–F), and “catalytic activity” in molecular function (MF, Figures 5G–I). The most significantly enriched GO terms for DAS genes are shown in Figures 6A–I. As the temperature decreased, the “response to abiotic stimulus” item of BP increased significantly, which is consistent with the GO terms of DEGs.

**Figure 7:**
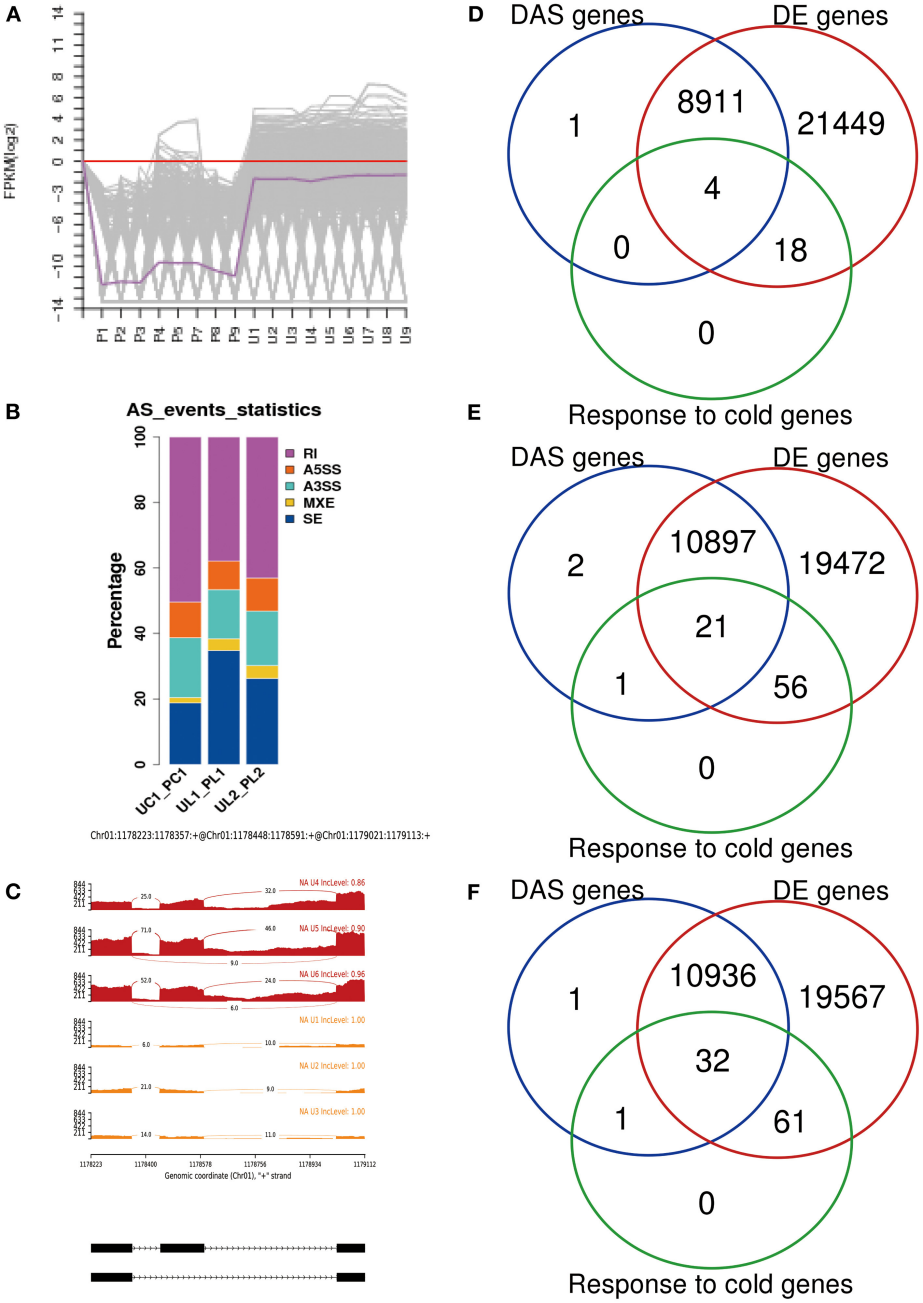
Changes in DEGs and DAS genes in response to cold stress. **(A)** One subclass of differentially expressed genes (DEGs) clustering in *P. ussuriensis* compared with *P. trichocarpa* in response to cold stress identified by RNA sequencing analysis. **(B)** AS events analysis between *P. trichocarpa* and *P. ussuriensis* UC1_PC1: AS percentage statistical analysis at 25°C in *P. trichocarpa* compared with *P. ussuriensis*; UL1_PL1: AS percentage statistical analysis at 3°C in *P. trichocarpa* compared with *P. ussuriensis*; UL2_PL2: AS percentage statistical analysis at −3°C in *P. trichocarpa* compared with *P. ussuriensis*. **(C)** A model pattern example from the IGV view of Potri.001G016200 alternative splicing (ES) type. **(D)** The Venn view of DAS genes, DE genes, and genes response to cold at 25°C. **(E)** The Venn view of DAS genes, DE genes, and genes response to cold at 3°C. **(F)** The Venn view of DAS genes, DE genes, and genes response to cold at −3°C.

**Figure 8:**
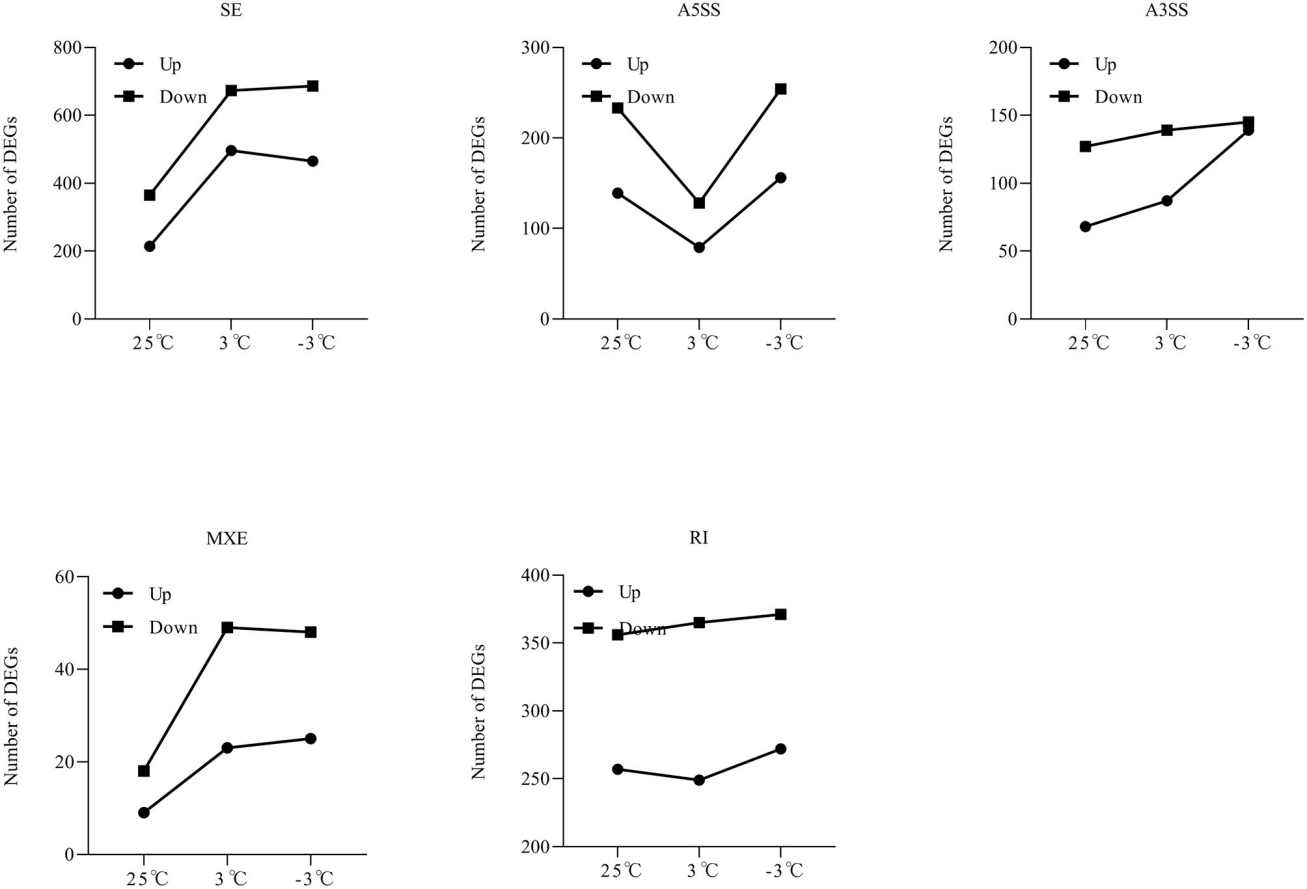
The dynamics of AS types under cold stress in *P. ussuriensis* compared with *P. trichocarpa*.

**Figure 9:**
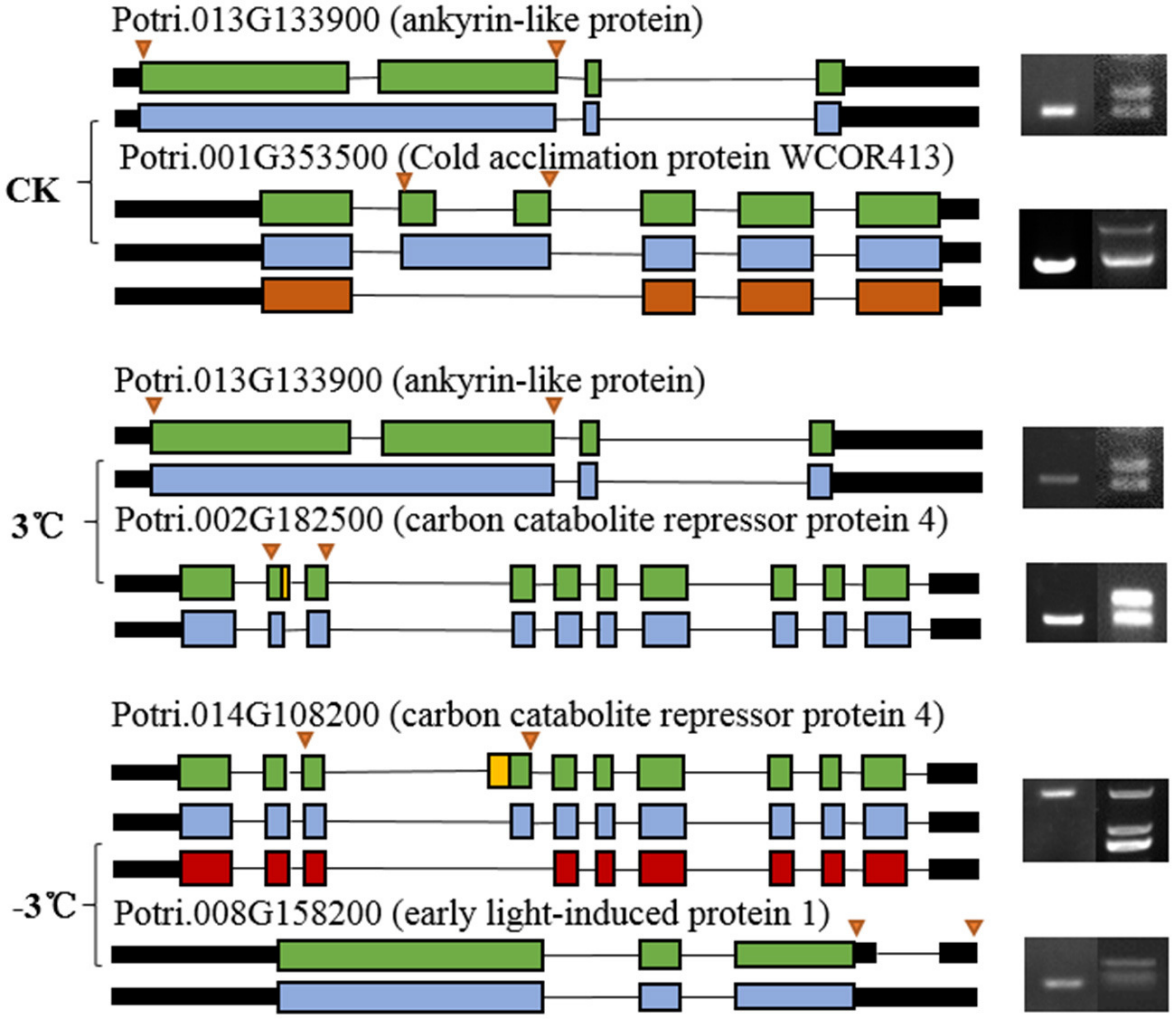
Characteristic of randomly selected AS events and validation of randomly selected alternative splicing events detected by RNA sequencing using reverse transcription-PCR (RT-PCR). The arrow on the left side indicates the position of PCR primers used for RT-PCR. The electropherogram on the right side shows the alternatively spliced product bands in *P. trichocarpa* and *P. ussuriensis*.

**Figure 10:**
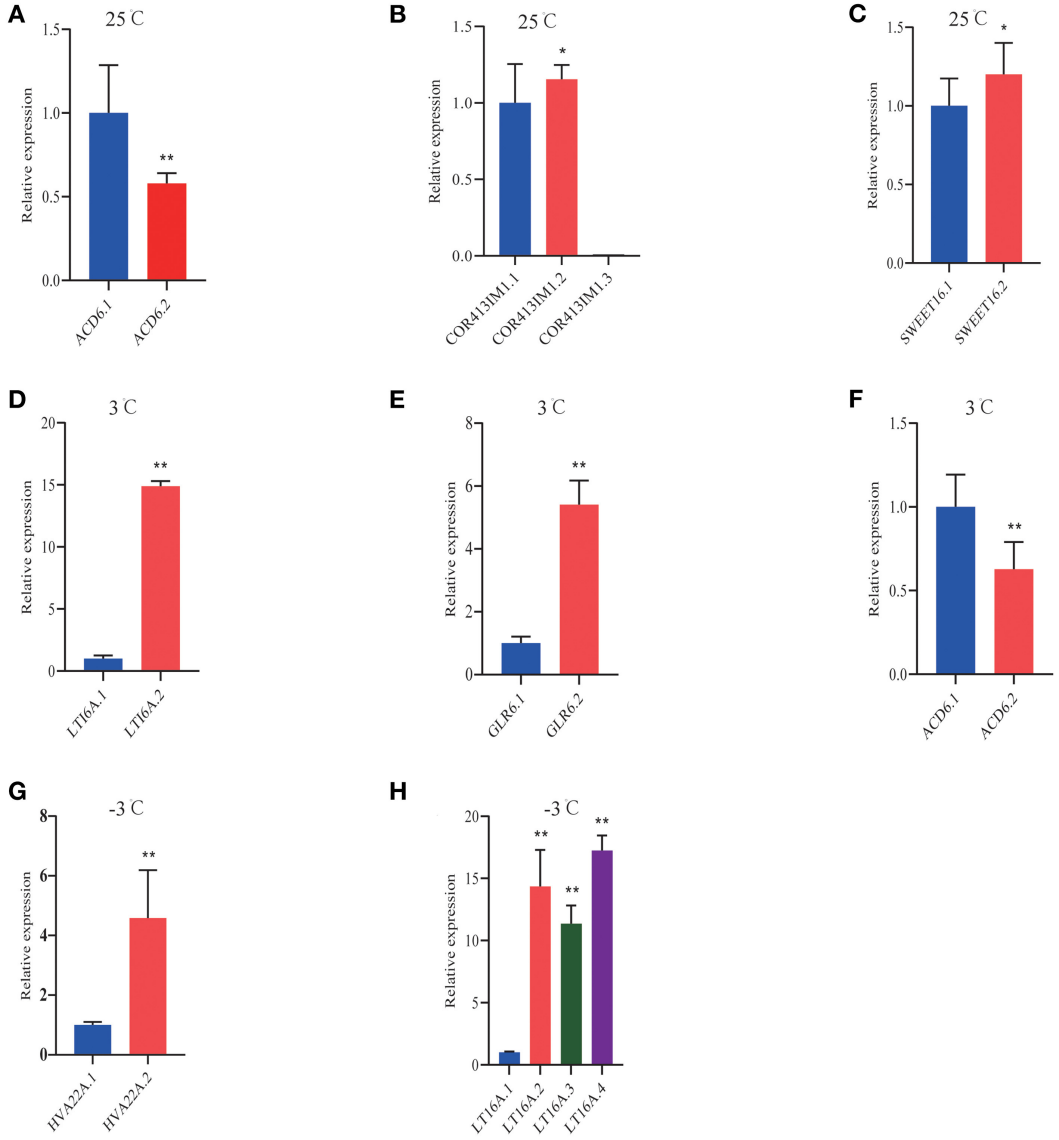
QRT-PCR analysis of DAS isoforms response to cold stress in *P. ussuriensis* compared with *P. trichocarpa*. **(A–C)** DAS genes at 25°C. **(D–F)** DAS genes at 3°C. **(G,H)** DAS genes at −3°C.

From the RNA-Seq data, U1–U9 samples were classified into six subclasses of genes that were upregulated or downregulated in *P. ussuriensis* compared with *P. trichocarpa* (P1–P9 samples) in response to cold stress with different patterns of expression (Supplementary Figure S1). For example, genes of subclass 3 in *P. ussuriensis* were upregulated compared with that in *P. trichocarpa* (Figure 7A). These upregulated genes included genes involved in calcium signaling, such as calmodulin (CAM), calcium-dependent protein kinase (CPK), and CBL-interacting protein kinase (CIPK) (Table 3), and many mitogen-activated protein kinase (MAPK) cascades (Supplementary Data S4). Additionally, a 9-*cis*-epoxycarotenoid dioxygenase (*NCED*) gene encoding the key enzyme of abscisic acid (ABA) biosynthesis was upregulated under cold stress (Table 3). Many peroxidase (POD) genes were also upregulated in *P. ussuriensis* (Table 3). We also used the RNA-Seq data to identify genes that were DAS between contrast poplar species. In total, 2,785 (IR, 50.4%; A5SS, 10.9%; A3SS, 18.2%; MXE, 1.7%; ES, 18.8%), 3,613 (IR, 37.9%; A5SS, 8.7%; A3SS, 15.%; MXE, 3.5%; ES, 34.8%), and 3,408 (IR, 43.1%; A5SS, 10.9%; A3SS, 16.5%; MXE, 4.%; ES, 26.3%) DAS genes were identified at 25, 3, and −3°C, respectively, in *P. ussuriensis* compared with that in *P. trichocarpa* by RNA-seq (Figure 7B). MATS identified the DAS events in the two species under different temperature conditions. A model pattern example from IGV view of Potri.001G016200 alternative splicing (ES) type is shown in Figure 7C. Venn analysis revealed a substantial overlap among the DEGs, DAS genes, and the cold-responsive DEGs identified in *P. ussuriensis* compared with *P. trichocarpa* (Figures 7D–F). About half of the DEGs were DAS genes (Figures 7D–F), of which 18.1, 28.2, and 35.1% DEGs at 25, 3, and −3°C, respectively, in response to cold stress were DAS genes (Figures 7D–F); these genes with significant expression (*p* < 0.05;|log2 (FC)|>1) are shown in Table 4 based on GO. We found that several DEGs related to cold stress, such as cold-regulated inner membrane protein 1 (CORIMP1, Potri.001G353500) at 25°C, low-temperature- and salt-responsive proteins (LTI6A, Potri.013G001600 and CORIMP1Potri.001G353500) at 3°C, and late elongated hypocotyl (LHY, Potri.014G106800), low-temperature-induced integral membrane protein (LTI6A, Potri.013G001600), and calcium-binding protein (CML, Potri.002G182500, Potri.014G108200) at −3°C in *P. ussuriensis* (Table 4) had more isoforms based on *P. trichocarpa* genome. Additionally, we investigated the dynamics of different AS types under cold stress; the result showed that the number of up and downregulated DEGs showing an upward trend as the temperature goes down in almost all AS types. In contrast, more genes were downregulated than upregulated in *P. ussuriensis* compared with *P. trichocarpa* (Figure 8). RT-PCR validation revealed more than one isoform for these genes (Figure 9). Meanwhile, the qRT-PCR result indicated that some genes that generated more transcripts were responsive to cold stress (Figure 10). We also randomly selected some DEGs using qRT-PCR to validate the reliability of the RNA-seq data. The result showed that the qRT-PCR was consistent with RNA-seq data (Supplementary Figure S2).

**Table 3:** Partial differentially expressed genes (DEGs) response to cold in *P. ussuriensis* compared with *P. trichocarpa*.

**25°C**			**+3°C**			**−3°C**		
**Gene ID**	**Annotation**	**Log_**2**_FoldChange**	**Gene ID**	**Annotation**	**Log_**2**_FoldChange**	**Gene ID**	**Annotation**	**Log_**2**_FoldChange**
Potri.001G138000	*CAM*	3.2		*CAM*	1.5	Potri.T124900	*CAM3*	1.5
Potri.007G042900	*CAM1*	2.0	Potri.T124900	*CAM3*	1.3	Potri.002G047300	*CAM*	1.4
Potri.005G052800	*CAM8*	1.8				Potri.001G138000	*CAM*	1.4
Potri.T124900	*CAM3*	1.7						
Potri.006G065900	*CAM3*	1.5						
Potri.009G168700	*CPK*	1.6	Potri.009G168700	*CPK*	3.6	Potri.009G168700	*CPK*	2.7
Potri.016G06570	*CPK*	1.2	Potri.004G015500	*CPK*	1.4	Potri.004G015500	*CPK*	1.3
Potri.004G015500	*CPK*	1.2				Potri.016G065700	*CPK*	1.2
Potri.018G122300	*CIPK*	1.4	Potri.018G108500	*CIPK*	1.3	Potri.016G133900	*CIPK*	1.4
Potri.018G108500	*CIPK*	1.2	Potri.018G122300	*CIPK*	1.3	Potri.017G118000	*CIPK*	1.2
Potri.009G152200	*NCED*	−1.8	Potri.001G393800	*NCED*	2.1	Potri.001G393800	*NCED*	1.3
			Potri.011G112400	*NCED*	1.5	Potri.011G112400	*NCED*	2.2
			Potri.019G093400	*NCED*	1.1			
Potri.010G156400	*CAB*	6.1	Potri.010G156400	*CAB*	9.1	Potri.010G156400	*CAB*	8.0
Potri.002G222800	*CAB*	4.5	Potri.002G222800	*CAB*	3.6	Potri.002G222800	*CAB*	2.8
Potri.008G198900	*CAB*	1.1	Potri.016G011300	*CAB*	3.3	Potri.016G011300	*CAB*	1.7
Potri.010G155400	*CAB*	−1.9	Potri.014G162100	*CAB*	2.6	Potri.010G155400	*CAB*	−1.9
			Potri.T137800	*CAB*	−1.4			
			Potri.010G155400	*CAB*	−2.1			
Potri.013G154400	*POD*	7.2	Potri.012G000300	*POD*	7.7	Potri.012G000300	*POD*	7.7
Potri.007G122100	*POD3*	5.5	Potri.007G122100	*POD*	4.6	Potri.017G038000	*POD3*	5.5
Potri.017G064100	*POD73*	5.4	Potri.017G038000	*POD3*	4.3	Potri.008G099900	*POD*	4.7
Potri.017G038000	*POD3*	4.3	Potri.011G062300	*POD19*	3.2	Potri.007G122100	*POD3*	4.3
Potri.T163100	*POD*	4.3	Potri.001G145800	*POD20*	3.1	Potri.008G022700	*POD*	3.7
Potri.013G083600	*POD4*	3.7	Potri.001G145800	*POD18*	3.1	Potri.013G154400	*POD*	3.4
Potri.012G006800	*POD5*	3.0	Potri.002G018000	*POD64*	2.8	Potri.016G125000	*POD*	3.4
Potri.010G036100	*POD6*	2.8	Potri.013G154400	*POD*	2.3	Potri.011G062300	*POD19*	2.9
Potri.016G132900	*POD*	2.6	Potri.003G214900	*POD15*	2.0	Potri.017G064100	*POD73*	2.8
Potri.018G015500	*POD18*	2.6	Potri.010G036100	*POD6*	1.9	Potri.018G015500	*POD18*	2.7
Potri.001G145800	*POD20*	2.6	Potri.003G214800	*POD15*	1.7	Potri.010G175100	*POD7*	2.6
Potri.001G013000	*POD*	2.1	Potri.001G013000	*POD*	1.6	Potri.001G145800	*POD20*	2.6
Potri.003G214900	*POD15*	2.1	Potri.017G064100	*POD73*	1.6	Potri.010G036100	*POD6*	2.2
Potri.015G003500	*POD5*	2.0	Potri.004G052100	*POD15*	1.5	Potri.003G214800	*POD15*	2.0
Potri.001G011500	*POD15*	1.9	Potri.003G214700	*POD15*	1.2	Potri.001G013000	*POD*	2.0
Potri.011G062300	*POD*	1.9				Potri.013G083600	*POD4*	1.6
Potri.002G018000	*POD64*	1.8				Potri.002G065300	*POD12*	1.6
Potri.003G214800	*POD15*	1.8				Potri.003G214700	*POD15*	1.5
Potri.006G267400	*POD18*	1.3				Potri.006G267400	*POD18*	1.2
Potri.007G053400	*POD73*	1.2						

**Table 4:** Differential alternative splicing events in response to cold in *P. ussuriensis* compared with *P. trichocarpa*.

**25°C**				**+3°C**				**−3°C**			
**Gene ID**	**AS type**	**Annotation**	**Log2FoldChange**	**Gene ID**	**AS type**	**Annotation**	**Log2FoldChange**	**Gene ID**	**AS type**	**Annotation**	**Log2FoldChange**
Potri.013G133900	RI	*ACD6*	−2.9	Potri.003G017500	ES	*RECQ*	1.8	Potri.013G133900	RI	*ACD6*	−3.5
Potri.001G353500	ES, RI	*COR413IM1*	−1.7	Potri.013G001600	A3SS	*LTI6A*	−1.4	Potri.009G078500	ES	HVA22A	−1.9
Potri.013G014400	RI	*SWEET16*	−1.6	Potri.008G158200	RI	*ELIP*	−2.4	Potri.014G106800	A3SS, A5SS, MXE, RI, ES	*LHY*	2.2
				Potri.005G070900	ES	DGK	−2.6	Potri.013G001600	A3SS, A5SS, ES	*LTI6A*	−2.9
				Potri.008G161600	ES	EO	1.3	Potri.018G004200	ES	HIBCH	−6.0
				Potri.005G246000	ES, A5SS	PLD	−1.4	Potri.013G014400	A3SS, RI, ES	SWEET16	−1.9
				Potri.002G230000	ES	GLR6	1.5	Potri.004G230100	A3SS, ES	VRN1	−1.6
				Potri.017G041700	A3SS	ISPS	1.4	Potri.001G024900	A3SS, ES	ANN4	1.5
				Potri.013G133900	RI,	ACD 6	−3.3	Potri.002G182500	A3SS, RI, ES	CML	1.8
				Potri.002G182500	A3SS	CML	1.6	Potri.014G108200	A3SS, ES	CML	1.4
				Potri.001G353500	A3SS, MXE, RI, ES	CORIM1	−1.4	Potri.005G070900	ES	DAG	−1.6
								Potri.008G158200	RI	ELIP1	−1.5
								Potri.005G196700	A5SS, ES	GI	−2.1

### Cold-induced as in Splicing Factors (SFs) and Transcription Factors (TFs)

**Figure 11:**
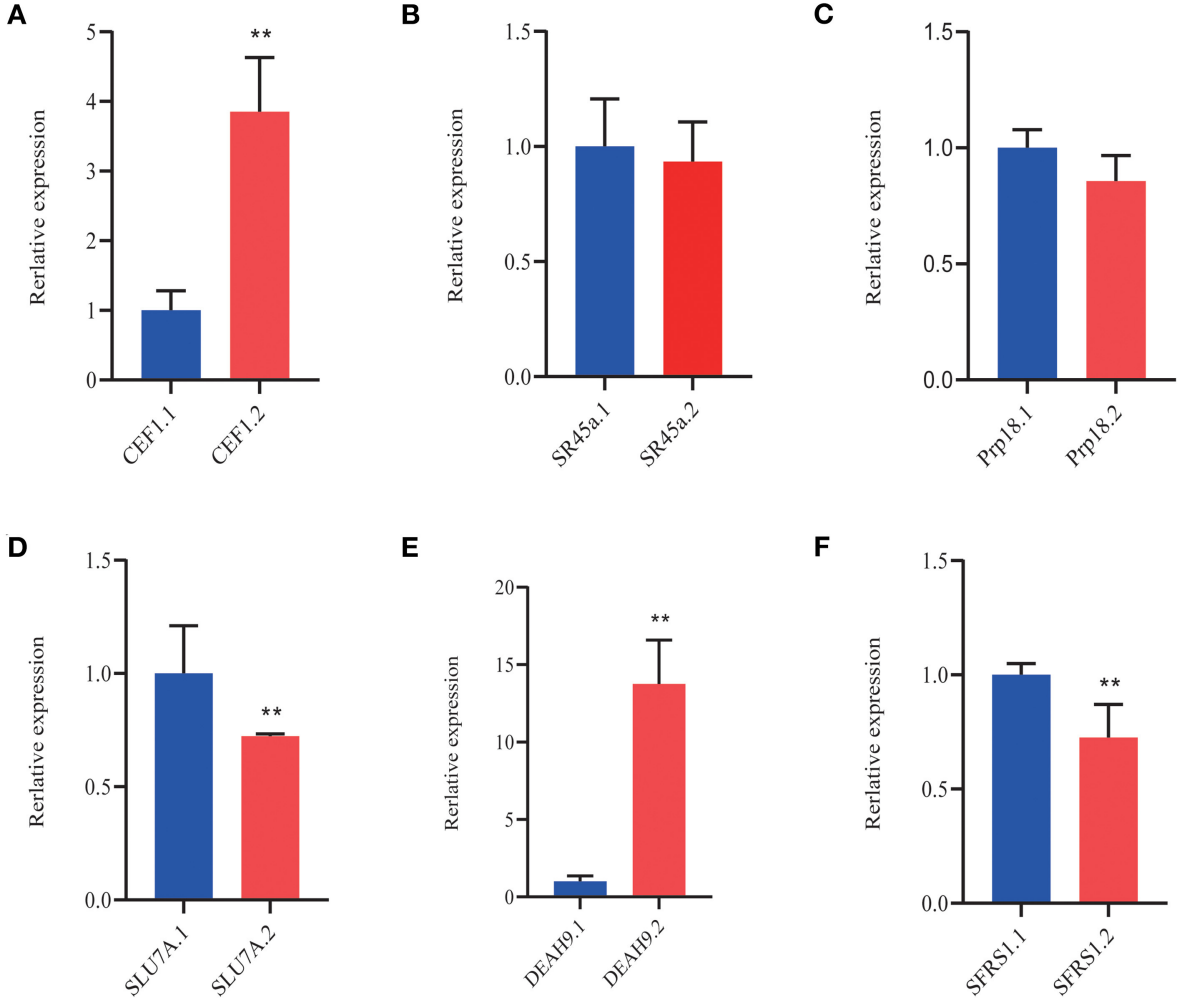
QRT-PCR analysis of DAS isoforms of splicing-associated factors in *P. ussuriensis* compared with *P. trichocarpa*. **(A)** CEF1. **(B)** SR45a. **(C)** Prp18. **(D)** SLU7A. **(E)** DEAH9. **(F)** SFRS1.

The splice site of pre-mRNA was recognized by SFs that recruit the spliceosome to remove the introns away (Chen and Moore, 2015). A number of SFs reported the functions in diverse environmental conditions (Laloum et al., 2018). We focused on the SFs that probably regulated the AS of downstream genes by different spliceosome complexes under a cold condition. The AS events occurred in multiple SFs, including small nuclear ribonucleo proteins (snRNPs), such as spliceosome-associated proteins (DEAH box helicase 1, YT521-B, LSM, AAR2, SART-1, and Prp series; Table 4, *p* < 0.05;|log2 (FC)|> 1). At 25°C, only two SFs were differentially expressed; five SFs, including the two SFs at 25°C, were differentially expressed at 3°C. Many SFs, including SFs of the two temperatures (25 and 3°C), were also responsive at −3°C (Table 4). Most importantly, all the SFs were upregulated. As shown in Figure 11, the induced SFs, such as CEF1, SR45a, Prp18, SLU7A, DEAH9, and SFRS1, have more AS isoforms in *P. ussuriensis* compared with *P. trichocarpa*. In short, more pre-mRNAS Fs responded in *P. ussuriensis* than that in *P. trichocarpa* as the temperature decreased.

**Figure 12:**
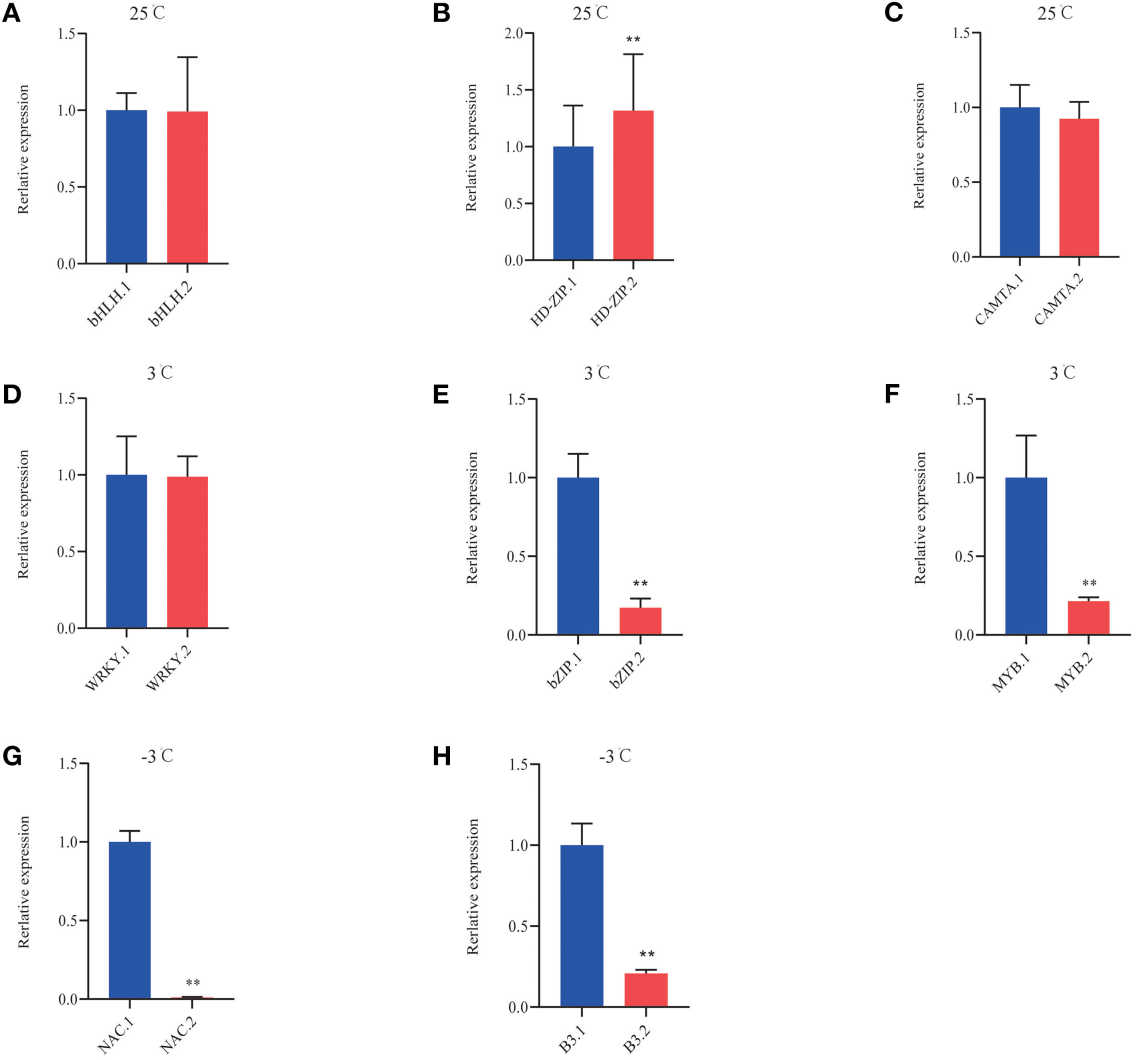
QRT-PCR analysis of DAS isoforms of TFs in *P. ussuriensis* compared with *P. trichocarpa*. **(A–C)** DAS genes of TFs at 25°C. **(D–F)** DAS genes of TFs at 3°C. **(G,H)** DAS genes of TFs at −3°C.

Analysis based on the reference genome revealed AS of numerous TFs. Table 3 shows significantly induced differentially expressed TFs in *P. ussuriensis* compared with *P. trichocarpa*. Under normal conditions, AS events of these TFs were different between the two poplar species and identified, such as WRKY, MYB, B3, and CAMTA. More and more AS events happened as the temperature lowered (Table 5). We selected some differentially expressed TFs that likely related to cold stress to conduct qRT-PCR analysis; the result showed that the isoforms of bHLH, HD-ZIP, CAMTA, WRKY, bZIP, MYB, NAC, and B3 TFs were differentially expressed in *P. ussuriensis* compared with *P. trichocarpa* (Figure 12).

**Table 5:** Expression change of splicing-associated factors in *P. ussuriensis* compared with *P. trichocarpa* at different temperatures.

**25°C**				**+3°C**				**−3°C**			
**Gene ID**	**AS type**	**Annotation**	**Log_**2**_FoldChange**	**Gene ID**	**AS type**	**Annotation**	**Log_**2**_FoldChange**	**Gene ID**	**AS type**	**Annotation**	**Log_**2**_FoldChange**
Potri.015G112800		DEAH1	5.6	Potri.016G020200		PRO8	2.4	Potri.015G112800		DEAH1	8.6
Potri.016G020200		PRO8	1.7	Potri.015G112800		DEAH1	2	Potri.016G020200		PRO8	3.5
				Potri.001G147100		RES	1.6	Potri.009G041300		RBM22	2.3
				Potri.010G093100	RI	CEF-1	1.2	Potri.010G093100	RI	CEF1	1.9
								Potri.016G062500	A3SS	SCL30A	1.8
								Potri.009G041700	A5SS	SR45a	1.8
								Potri.006G197000		SCL30A	1.7
								Potri.012G012800		SF3A3	1.6
								Potri.007G070700	A3SS; RI	SF3A1	1.6
								Potri.001G147100		RES	1.5
								Potri.007G045900	A5SS; RI	Prp18	1.4
								Potri.004G076600		CTNNBL	1.4
								Potri.012G064100		SLU7-A	1.4
								Potri.015G048200	A3SS	SLU7-A	1.3
								Potri.012G133600	ES	RS40	1.3
								Potri.009G149600		SFU2af	1.3
								Potri.017G060200		ISY1	1.3
								Potri.004G039000	ES	SF3B2	1.3
								Potri.004G052700	ES	DEAH9	1.3
								Potri.002G153600		RS40	1.3
								Potri.003G087200		RES	1.3
								Potri.005G138200		SFRS16	1.3
								Potri.T134200	ES	SFRS1	1.2
								Potri.011G098100		RBM17	1.2
								Potri.006G000200		cwf25	1.2
								Potri.010G213300		PRP3	1.2
								Potri.005G024600	A3SS	SFRS1	1.2
								Potri.T125700		SC35	1.2
								Potri.005G210600		Prp18	1.2
								Potri.013G135300		PRP1	1.2
								Potri.008G168000	A5SS, MXE; ES	SF3B5	1.2
								Potri.005G258000		SFU2af	1.2
								Potri.019G039900		Prp19	1.2
								Potri.005G105700	A5SS; RI; ES	SR45a	1.2
								Potri.002G175000	A3SS; ES	RSP31	1.2
								Potri.014G101800	A5SS; ES	RS31	1.2

## Discussion

**Figure 13:**
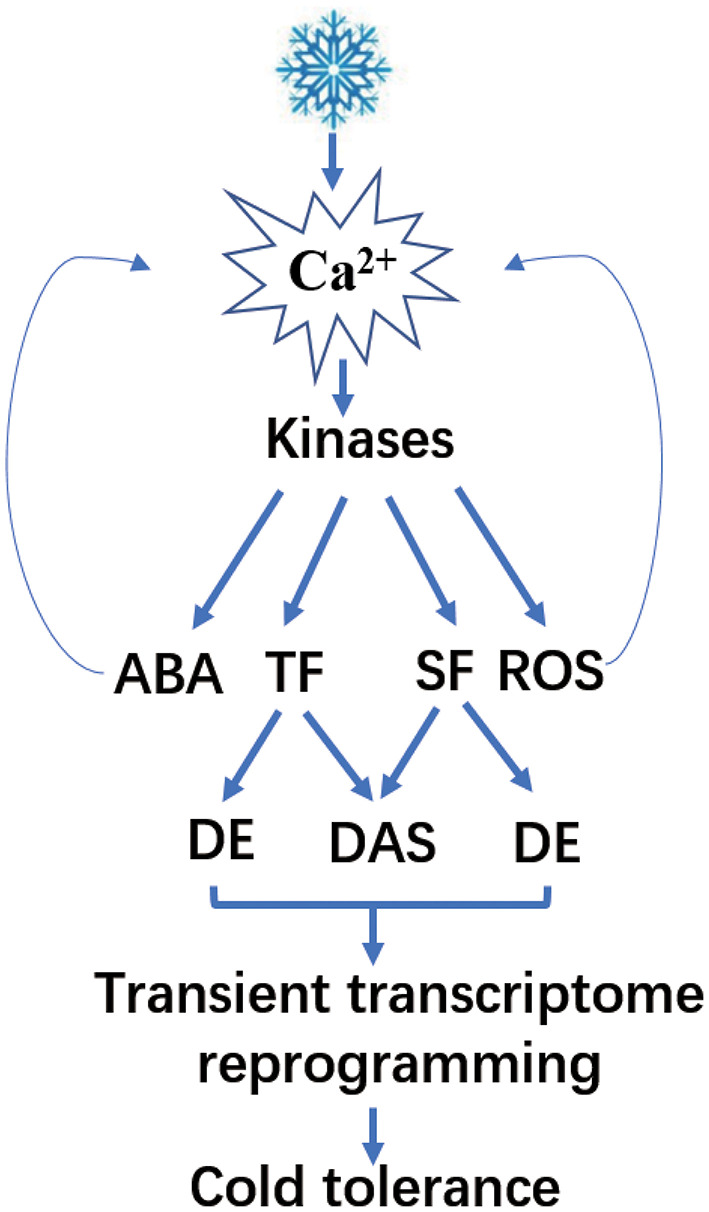
A model for the cold-signaling pathway and gene regulation in poplar.

Plants cope with adverse environmental conditions by reprogramming the gene expression and metabolism in a strict manner (Li et al., 2019). Although the expression of genes in some plant species under cold stress has been studied extensively, pre-mRNA splicing during the transcriptional changes in response to cold is still not clear in poplar species. In the current study, the result showed that the *P. ussuriensis* had more cold tolerance than *P. trichocarpa*, probably two poplar species have different abilities to deal with a cold condition. We used SMRT sequencing and RNA-Seq (Illumina) for the first time for a global survey of AS in two poplar species under cold stress. The PacBio Iso-Seq data revealed that 97.66 and 97.51% of the isoforms detected were mapped reads based on the reference genome of *P. trichocarpa*, and 1.8 and 1.02% were multi-mapped to the genome (Table 6; Figure 13).

**Table 6:** Comparison of alternative splicing (AS) events in differentially expressed TFs of two poplar species by the PacBio sequencing data, respectively.

**25°C**				**+3°C**				**−3°C**			
**Gene ID**	**AS type**	**Annotation**	**Log_**2**_FoldChange**	**Gene ID**	**AS type**	**Annotation**	**Log_**2**_FoldChange**	**Gene ID**	**AS type**	**Annotation**	**Log_**2**_FoldChange**
Potri.001G063000	A5SS	bHLH	4.3	Potri.001G283300	A3SS	ERF	5.8	Potri.005G184400	A5SS	B3	3.1
Potri.014G193800	A3SS	MYB	3.4	Potri.002G090600	A3SS, RI	bHLH	4.3	Potri.004G155800	MXE	CAMTA	4.0
Potri.012G016000	A3SS	NAC	2.1	Potri.001G395000	ES	MYB	5.3	Potri.014G106800	MXE	MYB	2.2
Potri.012G118300	A3SS	ERF	3.9	Potri.001G035500	A3SS, ES	WRKY	9.4	Potri.006G024000	RI	MYB	3.8
Potri.006G072200	A5SS	WRKY	2.6	Potri.001G010700	A3SS	WRKY	10.6	Potri.002G148000	RI	NAC	2.7
Potri.016G102100	A5SS	HD-ZIP	3.5	Potri.001G259100	A3SS,MXE, ES	NAC	5.9	Potri.001G184200	RI	WRKY	3.8
Potri.005G184400	A5SS	B3	2.4	Potri.001G056700	A3SS, ES	B3	8.8	Potri.002G102100	RI	ERF	2.6
Potri.004G155800	ES	CAMTA	3.4	Potri.002G002200	A3SS,A5SS	MYB	4.6	Potri.012G118300	ES	ERF	3.1
				Potri.001G063000	A5SS	WRKY	8.3	Potri.004G155800	ES	CAMTA	4.0
				Potri.002G225600	A5SS	HSF	3.8	Potri.007G119700	ES	bHLH	2.5
				Potri.001G075600	A5SS	MYB	8.0	Potri.T152100	ES	MYB	6.0
				Potri.001G290100	A5SS	WRKY	5.8	Potri.001G291700	ES	WRKY	2.9
				Potri.001G159400	MXE, ES	HSF	6.8	Potri.011G150100	ES	B3	2.8
				Potri.002G182500	RI	ERF	4.0				
				Potri.001G017700	RI	bHLH	9.6				
				Potri.001G436200	RI	B3	4.8				
				Potri.001G217700	RI	WRKY	6.2				
				Potri.001G056700	RI	B3	8.8				
				Potri.001G114000	RI	bHLH	7.5				
				Potri.001G255100	RI	bZIP	6.0				
				Potri.001G385300	ES	NAC	5.3				
				Potri.001G133500	ES	HSF	7.4				
				Potri.001G240300	ES	MYB	6.0				

Plant cells sense cold stress through membrane rigidification (Chinnusamy et al., 2007). The rigidification subsequently activates mechanosensitive or ligand-activated calcium channels that lead to transient accumulation of Ca^2+^ in the cytosol. Studies have reported higher levels of calcium, which lead to signal amplification and act as the initial signals for cold stress response in plants (Vergnolle et al., 2005; Chinnusamy et al., 2007). Ca^2+^ and MAPK-signaling cascades play significant roles in the cold response pathway of Arabidopsis (Li et al., 2017; Zhao et al., 2017). In our study, *P. ussuriensis* exhibited more cold tolerance than *P. trichocarpa*. We found that calcium signaling-related genes, such as CAM, CPK, and CIPK, and many MAPK cascades were upregulated in *P. ussuriensis* at 25, 3, and −3°C compared with *P. trichocarpa*. Meanwhile, the Ca^2+^ content in *P. ussuriensis* increased more than that in *P. trichocarpa* under cold stress in a short time (3 h) at different temperatures (Figure 5A), which indicates that the cold response pathway is calcium dependent in poplar species. Subsequently, calcium signal amplification and a range of signals might be involved in cold stress signaling as reported (Chinnusamy et al., 2007).

First, the abovementioned cytoplasmic Ca^2+^ is sensed by calcium sensors, such as calcium-dependent protein kinases (CPKs), which interact with downstream-signaling components, including hormones, such as ABA and reactive oxygen species (ROS) (Lv et al., 2018). ROS also activate signal transduction pathways in response to biotic and abiotic stresses (Miller et al., 2010). As the temperature lowed, the H_2_O_2_ content was increased, and the increased level was greater in *P. trichocarpa* compared with that in *P. ussuriensis*. In our study, low temperature upregulated many POD genes in *P. ussuriensis*, which indicate the influence of ROS on cold stress regulation of gene expression. Besides, ROS can simulate Ca^2+^ accumulation that affects cold tolerance in plants (Chinnusamy et al., 2007). In response to cold stress, plants usually accumulate an increased amount of ABA. ABA accumulates in plants to regulate stress-responsive genes (Lv et al., 2018). ABA can also act as a secondary signal to change Ca^2+^ levels that finally influence cold signaling (Boudsocq and Sheen, 2013). In this study, the *NCED* gene encoding the key enzyme involved in ABA biosynthesis at low temperature was upregulated. Meanwhile, ABA-enhanced stress tolerance is associated with ROS (Lv et al., 2018), suggesting an intensive cross-talk among ABA, ROS, and Ca^2+^. Together, we conclude that Ca^2+^ signaling, ROS, and ABA might have led to cold tolerance in a short time. Secondly, researchers have identified the cold-responsive ICE-CBF pathway, which was critical to the regulation of cold-responsive transcriptome in plants. One such cold-responsive pathway involved the binding of ICE1 (Inducer of CBF expression 1) to the promoter of CBFs (C-repeat binding factors)-inducing expression; ICE1 enhances the expression of *COR* (cold-regulated) genes also during cold acclimation (Ritonga and Chen, 2020). However, the expression of these cold-responsive genes did not change in the two poplar species. Therefore, we speculated this pathway is not the main reason that *P. ussuriensis* had more cold tolerance than *P. trichocarpa*.

As reported, Ca^2+^-dependent kinases activate or repress TFs or SFs. These, in turn, regulate the transcription or AS of downstream genes, thereby driving cascades of cold-induced gene expression (Calixto et al., 2018). Differential expression of SFs is a significant factor that determines the changes in stress-induced AS (Punzo et al., 2020). In the present study, all the SFs identified *via* GO enrichment analysis were upregulated in *P. ussuriensis* compared with *P. trichocarpa*. Among these upregulated SFs, SC35, an arginine-serine-rich protein, is known to regulate plant development by modulating splicing; and transcription of a subset of genes was identified (Yan et al., 2017). Scarecrow-like (*SCL*) genes are involved in plant information transmission *via* signaling networks (Liu et al., 2017), and in the regulation of plant abiotic stresses, such as drought or salt stress (Golldack et al., 2013) were identified. The SF RS40, also an arginine-serine-rich protein, participates in miRNA biogenesis (Chen and Moore, 2015). Therefore, the role of SFs in cold-induced AS changes needs further investigation. Additionally, studies have reported numerous TFs that are subjected to AS and subsequently contribute to the regulation of gene expression. In this study, a lot of TFs were differentially expressed in *P. ussuriensis* compared with *P. trichocarpa* at different temperatures. Among these, a few TFs had isoforms in *P. ussuriensis* compared with *P. trichocarpa* even at room temperature. As the temperature decreased, the amount of AS in TFs increased in *P. ussuriensis*. It will be interesting to investigate the functions of the novel and already known cold-responsive TFs regulated by AS under cold stress.

Abiotic stress triggers AS events in plants (Pajoro et al., 2017; Laloum et al., 2018). These events regulate proteome diversity and gene expression to adopt an adverse environment (Thatcher et al., 2016). Therefore, the DAS events identified in our study under cold stress were not accidental. The extensive AS information identified here demonstrates the complexity of cold stress response. Studies based on the analysis of DEGs alone have significantly underestimated this regulatory mechanism. We also compared the AS events in two poplar species in response to cold stress. The comparison of the different AS types identified IR events in a large proportion, followed by A3SS in both types of poplars. Studies have reported IR as the most common splicing event in plant species, such as Arabidopsis (Marquez et al., 2012; Calixto et al., 2018) and cassava (Li et al., 2019). Calixto et al. (2018) reported differential splicing events very early under cold stress in Arabidopsis. The AS exhibited significant changes within only 40–60 min under cold stress (Calixto et al., 2018). To identify DAS, we investigated the transcriptional changes in two poplar species in response to cold stress after 3 h by MATS. We identified few DAS events in *P. ussuriensis* when compared with *P. trichocarpa*. Many DAS genes regulated by both transcription and AS were differentially expressed in *P. ussuriensis* compared with *P. trichocarpa*. Accelerated cell death 6 (*ACD6*, Potri.013G133900) was downregulated in *P. ussuriensis* and subjected to IR under different low-temperature conditions. *ACD6* encodes a transmembrane protein with intracellular ankyrin repeats, which positively controls cell death and defense (Lu et al., 2003; Yao and Greenberg, 2006). Yao and Greenberg reported that *ACD* can prevent plant chlorophyll breakdown that induces programmed cell death (PCD) (Yao and Greenberg, 2006). In this study, *ACD6* was downregulated. This might have happened because PCD was not activated in *P. ussuriensis* at any temperature conditions, and thus, *P. ussuriensis* exhibited higher cold tolerance than *P. trichocarpa*. LHY is a protein that functions in floral growth (Yon et al., 2016) and stress response (Adams et al., 2018). Studies have reported the influence of temperature changes on *LHY* transcript in Arabidopsis; moreover, AS in *LHY* was temperature-dependent (James et al., 2018a,b). In the present study, *LHY* (Potri.014G106800) was upregulated, and five types isoforms (A3SS, A5SS, MXE, RI, and ES) were identified at −3°C, which indicates the role of *LHY* in cold tolerance in poplar. Together, our results suggest that AS and DEGs play critical roles in the cold response. We have proposed a network (Figure 9) that reflects the rapid changes in DEGs and DAS genes during the cold response.

## Conclusions

In summary, SMRT-Seq and Illumina RNA-Seq revealed that poplar trees rapidly responded at the pre-mRNA alternative splicing (AS) stage, and AS regulated the transcript abundance to adjust to cold stress. Isoform abundance and rapid response in P. ussuriensis, indicate that the changes in AS transcripts might be the most element that resistant to cold stress compared with *P. trichocarpa* species. Splicing factors and transcription factors are likely important for the regulation of DEGs and DAS. Our findings will support further research on the functions of AS and the coordination between transcriptional and AS responses to confer cold tolerance.

## Data Availability Statement

The datasets presented in this study can be found in online repositories. The names of the repository/repositories and accession number(s) can be found in the article/Supplementary Material.

## Author Contributions

JY and CL conceived and planned the experiments. JY, WL, and LS performed the experiments. HL, YF, and ZW collected the materials. CY and AC analyzed the data. JY wrote the manuscript with support from all the authors. All authors contributed to the article and approved the submitted version.

## Funding

This work was supported by the National Natural Science Foundation of China (No. 31971671), the Innovation Project of State Key Laboratory of Tree Genetics and Breeding (Northeast Forestry University) (2021A02), and the Heilongjiang Touyan Innovation Team Program (Tree Genetics and Breeding Innovation Team).

## Conflict of Interest

The authors declare that the research was conducted in the absence of any commercial or financial relationships that could be construed as a potential conflict of interest.

## Publisher's Note

All claims expressed in this article are solely those of the authors and do not necessarily represent those of their affiliated organizations, or those of the publisher, the editors and the reviewers. Any product that may be evaluated in this article, or claim that may be made by its manufacturer, is not guaranteed or endorsed by the publisher.

## Supplementary Material

Supplementary Data S1 Differentially expressed genes (DEGs) between *Populus ussuriensis* and *Populus trichocarpa* identified by RNA sequencing analysis.

Supplementary Data S2 DEGs between *Populus ussuriensis* and *Populus trichocarpa* at 3°C identified by RNA sequencing analysis.

Supplementary Data S3 DEGs between *Populus ussuriensis* and *Populus trichocarpa* at −3°C identified by RNA sequencing analysis.

Supplementary Data S4 DEGs of cluster 3 in *Populus ussuriensis* compared with *Populus trichocarpa* identified by RNA sequencing analysis.

Supplementary Figure S1 Clustering of differentially expressed genes (DEGs) in *Populus ussuriensis* compared with *Populus trichocarpa* in response to cold stress identified by RNA sequencing analysis.

Supplementary Figure S2 Validation of randomly selected DEGs using quantitative real-time PCR (qRT-PCR).

Supplementary Table S1 Primers used for reverse transcription-PCR (RT-PCR).

Supplementary Table S2 Primers used for quantitative real-time PCR (qRT-PCR).

Supplementary Table S3 Primers used for qRT-PCR analysis of DAS isoforms response to cold stress in *P. ussuriensis* compared to *P. trichocarpa*.

Supplementary Table S4 Primers used for qRT-PCR analysis of DAS isoforms of splicing-associated factors in *P. ussuriensis* compared to *P. trichocarpa*.

Supplementary Table S5 Primers used for qRT-PCR analysis of DAS isoforms of TFs in *P. ussuriensis* compared to *P. trichocarpa*.
