# Supplementary figures and images for: PacBio and Illumina RNA Sequencing Identify Alternative Splicing Events in Response to Cold Stress in Two Poplar Species
Source: Front Plant Sci. 2021 Oct 7;12:737004. doi: 10.3389/fpls.2021.737004 (PMC8529222; doi:10.3389/fpls.2021.737004)

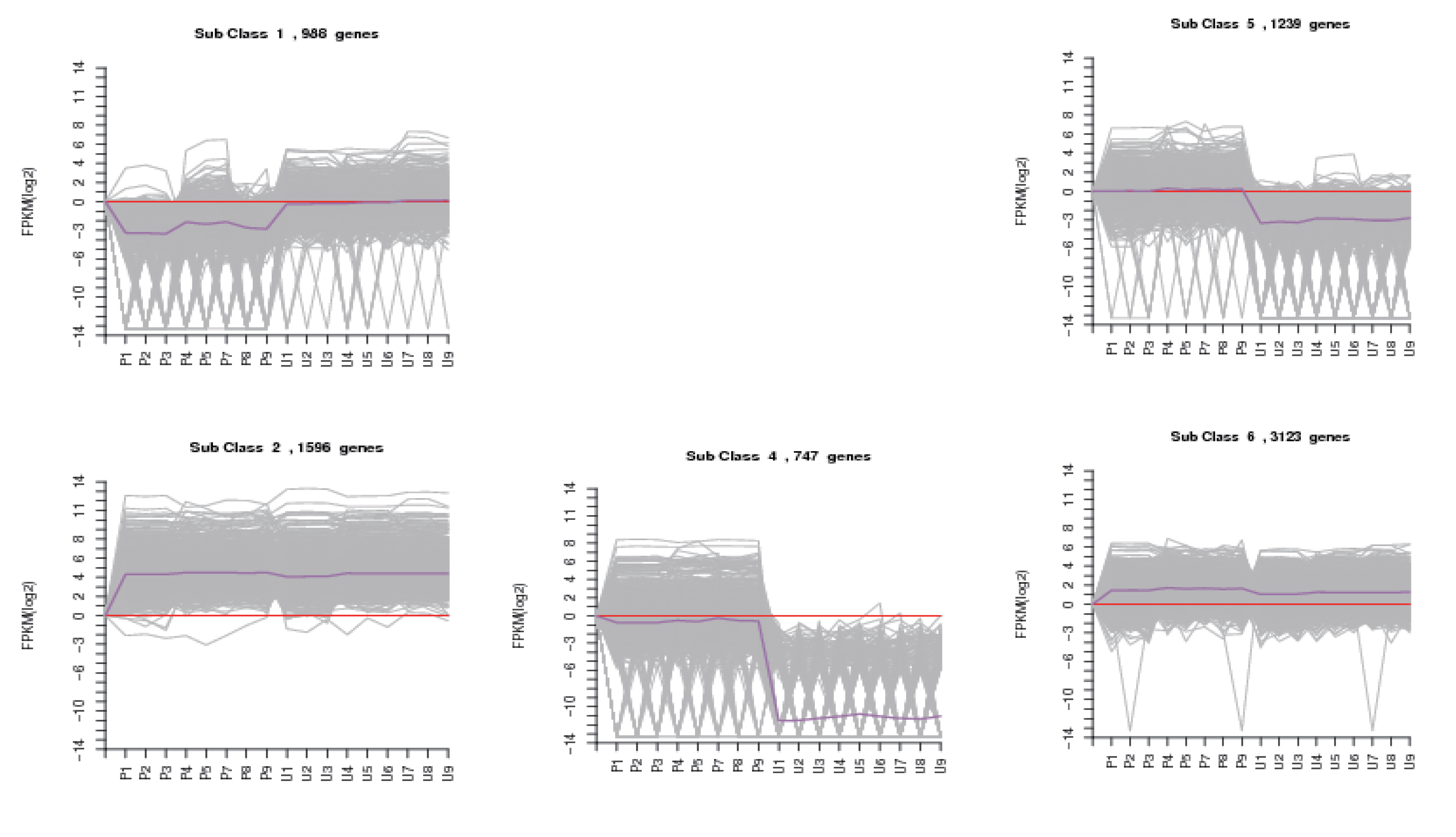

Supplement: Supplementary Figure S1 — Clustering of differentially expressed genes (DEGs) in Populus ussuriensis compared with Populus trichocarpa in response to cold stress identified by RNA sequencing analysis. [file Image_1.tiff]

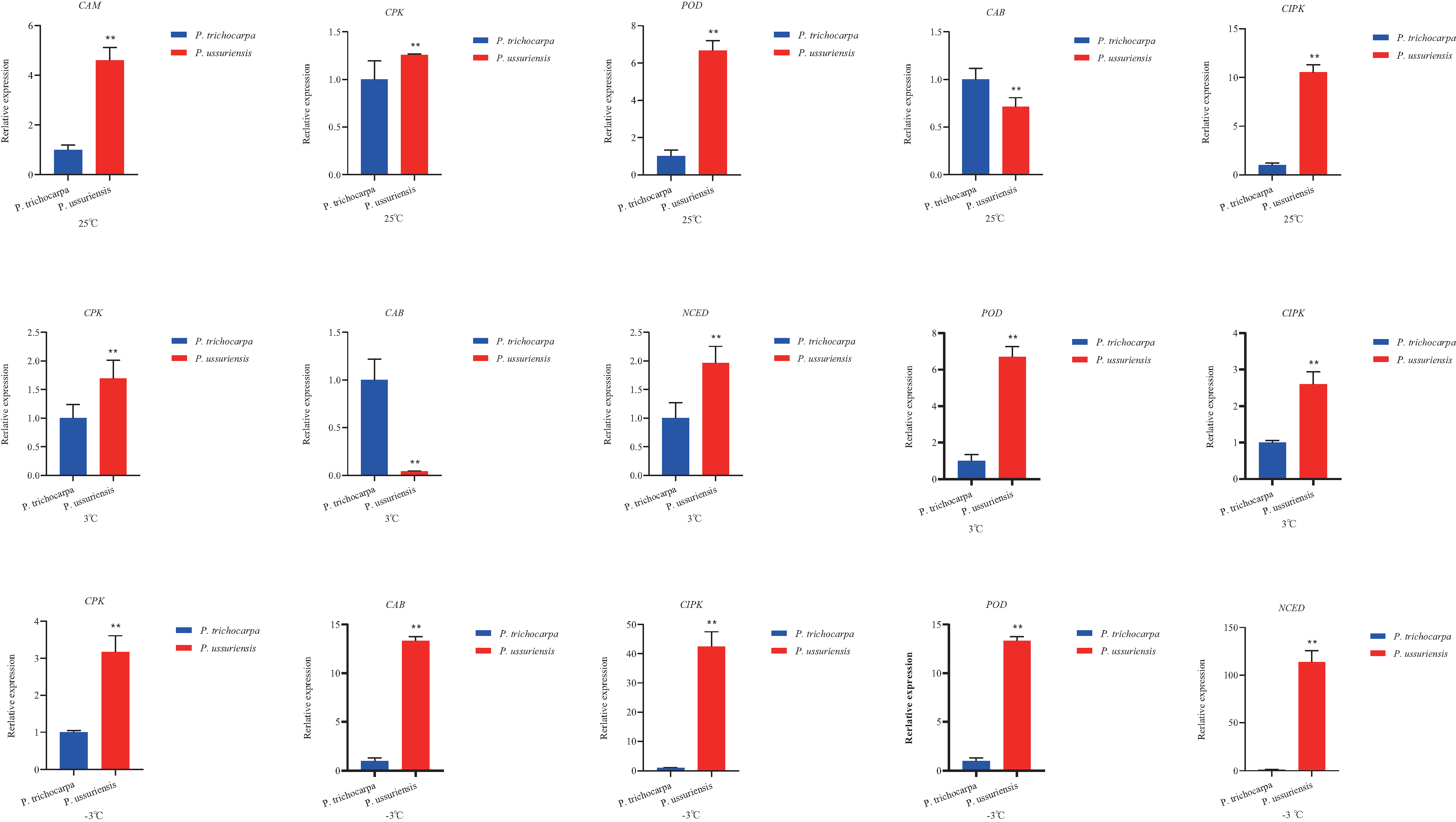

Supplement: Supplementary Figure S2 — Validation of randomly selected DEGs using quantitative real-time PCR (qRT-PCR). [file Image_2.tiff]
