# Supplementary material for: PacBio and Illumina RNA Sequencing Identify Alternative Splicing Events in Response to Cold Stress in Two Poplar Species
Source: Front Plant Sci. 2021 Oct 7;12:737004. doi: 10.3389/fpls.2021.737004 (PMC8529222; doi:10.3389/fpls.2021.737004)
Supplement: Supplementary Table S1 — Primers used for reverse transcription-PCR (RT-PCR). [file Table_1.docx]

Table S1 Primers for RT-PCR validation of the differential AS (DAS) events.

| Gene ID | Primer (5’→3’) | Length (bp) |
| --- | --- | --- |
| Ankyrin-like protein | F: GATTCCATTGACATGCAGTCTGGTC  R: CGTTTCATCTCTTCCTTCATATTGC | 1396 |
|  | F: GATTCCATTGACATGCAGTCTGGTC  R: CGTTTCATCTCTTCCTTCATATTGC | 1294 |
| Cold acclimation protein WCOR413 | F: TTGGTGCTGGCAAAAGGCACTGC  R: AATGCAGTCCAAATGCCATGCC | 135 |
|  | F: TTGGTGCTGGCAAAAGGCACTGC  R: AATGCAGTCCAAATGCCATGCC | 286 |
|  | F: TTGGTGCTGGCAAAAGGCACTGC  R: AATGCAGTCCAAATGCCATGCC | 0 |
| Ankyrin-like protein | F: GATTCCATTGACATGCAGTCTGGTC  R: CGTTTCATCTCTTCCTTCATATTGC | 1396 |
|  | F: GATTCCATTGACATGCAGTCTGGTC  R: CGTTTCATCTCTTCCTTCATATTGC | 1294 |
| Carbon catabolite repressor protein 4 | F: GAATTCTGGGTTGGAAATGAAG  R: GACAGGAATCGTGAGGAAAT | 298 |
|  | F: GAATTCTGGGTTGGAAATGAAG  R: GACAGGAATCGTGAGGAAAT | 266 |
| Carbon catabolite repressor protein 4 | F: GAATTCTGGGTTGGAAATGAAG  R: CCATTCCAATCACTGGATAAAGC | 306 |
|  | F: GAATTCTGGGTTGGAAATGAAG  R: CCATTCCAATCACTGGATAAAGC | 250 |
|  | F: GAATTCTGGGTTGGAAATGAAG  R: CCATTCCAATCACTGGATAAAGC | 112 |
| Early light-induced protein 1 | F: GCGTAATGACATCTGATGCTGAG  R: TGCGTGAATGGTTACAAGTCCG | 245 |
|  | F: GCGTAATGACATCTGATGCTGAG  R: TGCGTGAATGGTTACAAGTCCG | 329 |
