# Supplementary material for: PacBio and Illumina RNA Sequencing Identify Alternative Splicing Events in Response to Cold Stress in Two Poplar Species
Source: Front Plant Sci. 2021 Oct 7;12:737004. doi: 10.3389/fpls.2021.737004 (PMC8529222; doi:10.3389/fpls.2021.737004)
Supplement: Supplementary Table S2 — Primers used for quantitative real-time PCR (qRT-PCR). [file Table_2.docx]

Table S2 Primers for qRT-PCR validation of the differential expressed genes.

| Gene name | Primer (5’→3’) | Length (bp) |
| --- | --- | --- |
| CAM | F: GCACCAGAGCTGCTTCCGGAG  R: CCGTGTCTGCCTCCTTGATC | 184 |
| CPK.1 | F: GGTGTGATGCATAGAGATCTC  R: CTGCGCCGCAATACCTCAGG | 173 |
| CPK.2 | F: GTGGTGGTGGAGGAGGAGGAAG  R: CATTGTTGAAATCTCCCGTT | 206 |
| CPK.3 | F: ATGAAGATGGTGAAGCACCC  R: CACGATGATAAACACCGCGAC | 211 |
| CPK.4 | F: GGATGTTCAATGAATCCGATAC  R: CAGATGATCCGTTAACCGGTG | 206 |
| NCED | F: GGTGAGCTACATGGTCATTCGG  R: CAGTCTGAAGGTCACCAGAGG | 199 |
| CAB | F: CACGATATGTGAGCTCAAGG  R: CATCCTCTTTGTCTATCTTGA | 210 |
| POD.1 | F: CAGGCATATCCATAATGCTCC  R: GACTACAGCAGGGCAGGCCGC | 217 |
| POD.2 | F: CTAAAGGTTGTTGAGACACAG  R: TCCAATTGCTTACTTGTTGTG | 202 |
