# Supplementary material for: PacBio and Illumina RNA Sequencing Identify Alternative Splicing Events in Response to Cold Stress in Two Poplar Species
Source: Front Plant Sci. 2021 Oct 7;12:737004. doi: 10.3389/fpls.2021.737004 (PMC8529222; doi:10.3389/fpls.2021.737004)
Supplement: Supplementary Table S3 — Primers used for qRT-PCR analysis of DAS isoforms response to cold stress in P. ussuriensis compared to P. trichocarpa. [file Table_3.docx]

Table S3 Primers used for qRT-PCR analysis of DAS isoforms response to cold stress in *P. ussuriensis* compared to *P. trichocarpa*.

| Treatment | Gene name | Primers (5’→3’) | Length (bp) |
| --- | --- | --- | --- |
| 25℃ | *ACD6.1* | F1: CCGTATCATAGGTGATCACAAT  R1: CGATTTTCTGCAATATACCTG | 131 |
|  | *ACD6.2* | F2: TCGCAATATGAACGGGATTC  R2: ATGGAGACGTAATAACTCTGG | 190 |
|  | *COR413IM1.1* | F3: GGTGTTGGTGCTGGCAAAAGG  R3: CGGGAATAAAGAAAAAGAG | 161 |
|  | *COR413IM1.2* | F4: GGTGTTGGTGCTGGCAAAAG  R4: CGGGAATAAAGAAAAAGAG | 161 |
|  | *COR413IM1.3* | F5: ACTTCTATAGAATCCCATG  R5: AGAAGACCCACGATTGAAAC | 118 |
|  | *SWEET16.1* | F6: ACAGTGGTAGCAACGAAGAG  R6: CTAGGAAACCAAATTGGAAG | 270 |
|  | *SWEET16.2* | F7: GTGAGTTAGAATCTTCTCCC  R7: CTACATATGATCCAAAAC | 253 |
| 3℃ | *LTI6A.1* | F8: ATGTCAAGTACTAACTTC  R8: GCAATAAGGGCTGCTAACCTC | 233 |
|  | *LTI6A.2* | F9: GCGGAGTTTTGGATCTGC  R9: AACAAGAGATGAAGAAG | 117 |
|  | *GLR6.1* | F10: CTGCTTGGACGTGGTGTAG  R10: CAATCCGTGATGTTAATTGC | 151 |
|  | *GLR6.2* | F11: GAGAGAACACTCTGAGCACC  R11: GATTCTGCGATGTTGAGCTCG | 232 |
|  | *ACD6.1* | F12: CGGGATTCTTGATGATCTC  R12: GGAGACGTAATAACTCTGGC | 168 |
|  | *ACD6.2* | F13: TCGCAATATGAACGGGATTC  R13: GGAGACGTAATAACTCTGGC | 150 |
| -3℃ | *HVA22A.1* | F14: CTTCGTTTCACACCTTCAC  R14: CCCAAACCAAAAACAGGC | 220 |
|  | *HVA22A.2* | F15: CTTCGTTTCACACCTTCAC  R15: GCTAGAATGGAAGCACAT | 238 |
|  | *LTI6A.1* | F16: ATGTCAAGTACTAACTTCATAG  R16: CAGCAATAAGGGCTGCTAACCTC | *236* |
|  | *LTI6A.2* | F17: GCGGAGTTTTGGATCTGCTTG  R17: AACAAGAGATCATGAAGAAG | *117* |
|  | *LTI6A.3* | F18: GCGGAGTTTTGGATCTGCTTG  R18: CGGTACTGCGTCTCTCCAC | *136* |
|  | *LTI6A.4* | F19: CCTCAAGTTTGGTTGCGGG  R19: AACAAGAGATCATGAAGAAG | *136* |
