# Supplementary material for: PacBio and Illumina RNA Sequencing Identify Alternative Splicing Events in Response to Cold Stress in Two Poplar Species
Source: Front Plant Sci. 2021 Oct 7;12:737004. doi: 10.3389/fpls.2021.737004 (PMC8529222; doi:10.3389/fpls.2021.737004)
Supplement: Supplementary Table S4 — Primers used for qRT-PCR analysis of DAS isoforms of splicing-associated factors in P. ussuriensis compared to P. trichocarpa. [file Table_4.docx]

Table S4 Primers used for qRT-PCR analysis of DAS isoforms of splicing-associated factors in *P. ussuriensis* compared to *P. trichocarpa*.

| Treatment | Gene name | Primers (5’→3’) | Length (bp) |
| --- | --- | --- | --- |
| 3℃ | *CEF1.1* | F20: CAAGGAAGTTCCTTGAAAGG  R20: GTTGATGAAATAAAAGGAGA | 177 |
|  | *CEF1.2* | F21: CAAGGAAGTTCCTTGAAAGG  R21: GCAAGCCTCTTACCCC | 134 |
| -3℃ | *SR45a.1* | F22: GGTCAAGGTCCAGGTCCAG  R22: CAATTACCGTTCCTTCGGCGG | 135 |
|  | *SR45a.2* | F23: GGTCAAGGTCAAGGTCCAG  R23: CAATTACCGTTCCTTCGGCGG | 141 |
|  | *Prp18.1* | F24: GTAGGTTTGTTTCCAGATG  R24: GCTTCTAACTTACGTCCAATC | 179 |
|  | *Prp18.2* | F25: GTAGGTTTGTTTCCAGATG  R25: GAACTTCAGCATAACCTCTGC | 162 |
|  | *SLU7-A.1* | F26: GAGACTGTACTTCCTAGAAGCA  R26: CTTTAGTGGCCTCTTTACG | 217 |
|  | *SLU7-A.2* | F27: GAGACTGTACTTCCTAGAAGCA  R27: CTTTTTAAGCGCTTCAGC | 321 |
|  | *DEAH9.1* | F28: TCTATTTGGGTTTCTGG  R28: CAGCATGTCCCCTTCACTTG | 258 |
|  | *DEAH9.2* | F29: GGTGACCATGTTACATTCC  R29: CAGCATGTCCCCTTCACTTG | 159 |
|  | *SFRS1.1* | F30: GTGAGGGAATATGATTCCAAAC  R30: AGATGAAAGTGGAGATCTTG | 252 |
|  | *SFRS1.2* | F31: CAAGTCTCCTAAAAACAAATCC  R31: AGATGAAAGTGGAGATCTTG | 136 |
