# Supplementary material for: PacBio and Illumina RNA Sequencing Identify Alternative Splicing Events in Response to Cold Stress in Two Poplar Species
Source: Front Plant Sci. 2021 Oct 7;12:737004. doi: 10.3389/fpls.2021.737004 (PMC8529222; doi:10.3389/fpls.2021.737004)
Supplement: Supplementary Table S5 — Primers used for qRT-PCR analysis of DAS isoforms of TFs in P. ussuriensis compared to P. trichocarpa. [file Table_5.docx]

Table S5 Primers used for qRT-PCR analysis of DAS isoforms of TFs in *P. ussuriensis* compared to *P. trichocarpa*.

| Treatment | Gene name | Primers (5’→3’) | Length (bp) |
| --- | --- | --- | --- |
| 25℃ | *bHLH.1* | F32: ATGGAGAACAACCCTAGTTC  R32: CAGTGACAGTACTGGTTC | 156 |
|  | *bHLH.2* | F33: ATGGAGAACAACCCTAGTTC  R33: CCTTGAGCTTTGATGGGGT | 180 |
|  | *HD-ZIP.1* | F34: GACAATGGGGTAGTAAAGC  R34: CAGGCTTATAGCCCTCTG | 187 |
|  | *HD-ZIP.2* | F35: GACAATGGGGTAGTAAAGC  R35: CAAGTCCTGCAAAGTCTTCCC | 138 |
|  | *CAMTA.1* | F36: GTTTTCAAATCCATCTTGGAG  R36: GTCATCCCCTCCCCACAG | 184 |
|  | *CAMTA.2* | F37: GTCATGTTTGACAACAGGAAGG  R37: GTCATCCCCTCCCCACAG | 156 |
| 3℃ | *WRKY.1* | F38: ATGGAGAACAACCCTAGTTC  R38: GCAGTGACAGTACTGGTTC |  |
|  | *WRKY.2* | F39: ATGGAGAACAACCCTAGTTC  R39: CCTTGAGCTTTGAGATGGGGTA |  |
|  | *bZIP.1* | F40: ATGGGAGGTTATTCATCTAG  R40: CCAACCACTTTCCGATACAAC | 148 |
|  | *bZIP.2* | F41: ATGGGAGGTTATTCATCTAG  R41: TAACATTTGTTTGGAAAAG | 196 |
|  | *MYB.1* | F42: AATGGGACCATTGAGGGGG  R42: GAATCACCCAGGGAATGCC | 189 |
|  | *MYB.2* | F43: AGGTTGTTGTGAGCCCTCC  R43: CTAATCTCTCCAGTAAAAG | 124 |
| -3℃ | *NAC.1* | F44: GCTTCAAACTTATCCATAGG  R44: CTCGTACTGTTCCTGATCAC | 201 |
|  | *NAC.2* | F45: GCTTCAAACTTATCCATAGG  R45: CTGAGATGATGTCCGTTAG | 278 |
|  | *B3.1* | F46: GCAATCCTCCATCATTCAG  R46: CTGCATTTGATCACCGATGTTG | 341 |
|  | *B3.2* | F47: GAAAGATGTGGCTGAATATTG  R47: CTGCATTTGATCACCGATGTTG | 322 |
